# Training and experimental validation a novel anoikis- and epithelial‒mesenchymal transition-related signature for evaluating prognosis and predicting immunotherapy efficacy in gastric cancer

**DOI:** 10.7150/jca.106029

**Published:** 2025-01-06

**Authors:** Cheng Zeng, Chang Xu, Yuhan Wei, Fei Ma, Yue Wang

**Affiliations:** 1 Department of Medical Oncology, National Cancer Center/National Clinical Research Center for Cancer/Cancer Hospital, Chinese Academy of Medical Sciences and Peking Union Medical College, Beijing, 100021, China; 2 Department of Radiation Oncology, National Cancer Center/National Clinical Research Center for Cancer/Cancer Hospital, Chinese Academy of Medical Sciences and Peking Union Medical College, Beijing, 100021, China; 3 Cancer Institute, Xuzhou Medical University, Xuzhou, Jiangsu Province, 221004, China; 4 Department of Oncology, Wujin Clinical College of Xuzhou Medical University, Changzhou, Jiangsu Province, 213000, China; 5 Department of Oncology, Wujin Hospital Affiliated with Jiangsu University, Changzhou, Jiangsu Province, 213000, China

**Keywords:** Anoikis, Epithelial‒mesenchymal transition, Tumor microenvironment, Molecular pattern, Immunotherapy, Gastric cancer

## Abstract

Anoikis resistance and improper activation of epithelial‒mesenchymal transition (EMT) are critical factors in tumor metastasis and progression. Despite their interaction, the combined impact of anoikis and EMT on prognosis and immunotherapy in gastric cancer remains underexplored. In this study, we identified 354 anoikis- and EMT-related genes (AERGs) through Venn analysis and performed unsupervised clustering to classify gastric cancer patients into two molecular clusters: A and B. Molecular cluster A showed poor prognosis and an immunosuppressive tumor microenvironment, suggesting a "cold tumor" phenotype. Then, a novel AERG-related prognostic model comprising CD24, CRYAB, MMP11, MUC4, PRKAA2, SERPINE1, SKP2, and TP53 was constructed and validated, accurately predicting the 1-, 3-, and 5-year survival rates of gastric cancer patients. Multivariate analysis revealed that the AERG-related risk score was an independent prognostic factor (hazard ratio = 1.651, 95% confidence interval = 1.429-1.907,* P*<0.001). Further studies demonstrated that, compared to the high-risk group, the low-risk group exhibited higher CD8^+^ T cell infiltration, tumor mutational burden, immunophenoscores, and lower tumor immune dysfunction and exclusion scores, indicating potential sensitivity to immunotherapy. RT‒qPCR and immunohistochemical staining validated the expression levels of the model's molecular markers. Overall, our AERG-related model shows promise for predicting outcomes and guiding the selection of tailored and precise therapies for gastric cancer patients.

## Introduction

Gastric cancer is a highly heterogeneous malignant tumor with the fifth highest incidence and the third highest mortality rate worldwide^1^. Some individuals have already reached the progressive stage by the time they are diagnosed, and the prognosis is poor for patients with advanced stomach cancer^2^. Traditional chemotherapeutic drugs have entered a bottleneck, and the targeted drug trastuzumab has improved the survival rate of advanced gastric cancer patients with human epidermal growth factor receptor 2 (HER2) positivity. However, the proportion of HER-2-positive patients is only approximately 5%^3^, ^4^. Owing to their modest side effects and outstanding effectiveness, immune checkpoint inhibitors (ICIs) have provided hope for patients with advanced gastric cancer in recent years. Nonetheless, the response rate to ICIs is less than 30%, significantly limiting their widespread clinical use^5^. The tumor microenvironment (TME) is the internal environment of the tumor and is largely composed of tumor cells, stromal cells such as cancer-associated fibroblasts (CAFs), immune cells, cytokines, and the extracellular matrix (ECM)^6^. Inhibitory alteration of the TME is an essential factor affecting the efficacy of immunotherapy. Therefore, understanding TME characteristics can help screen the population for potential benefits of immunotherapy. Recently, several researchers have constructed prognostic models through transcriptomic analysis and screened people with high response rates to immunotherapy on the basis of molecular features or risk scores^7^-^9^.

Anoikis is a form of programmed cell death caused by the detachment of cells from the extracellular matrix, commonly seen when normal epithelial cells remain suspended for an extended period^10^. Anoikis is triggered mainly through both the intrinsic and extrinsic pathways. The intrinsic pathway promotes anoikis by upregulating proapoptotic molecules (Bad, Puma, Bik, Noxa, and Hrk) and downregulating antiapoptotic proteins of the Bcl-2 family, which leads to proteolytic hydrolysis of caspase-specific targets. The extrinsic pathway is triggered by the upregulation of Fas receptor expression^10^. Anoikis resistance (AR) is a characteristic of tumor metastasis that facilitates the movement of tumor cells through the circulatory system and into distant organs^11^. In gastric cancer, resistance to anoikis can promote angiogenesis and peritoneal metastasis through C/EBPβ-mediated PDGFB autocrine and paracrine signaling^12^. Increased AR is associated with poor prognosis in gastric cancer patients, as it enables tumor cells to evade apoptosis and contribute to metastatic spread^13^. Several researchers constructed anoikis-related risk model to predict the prognosis of gastric cancer patients and immunotherapy response^14^, ^15^.

Epithelial‒mesenchymal transition (EMT) is a biological process in which epithelial cells undergo transformation and acquire a mesenchymal phenotype. This transformation is characterized by a decrease in E-cadherin expression and an increase in N-cadherin, vimentin, and fibronectin expression^16^. In gastric cancer, abnormal activation of EMT leads to tumor migration and invasion, enhances the presence of tumor stem cells, increases resistance to chemotherapy, and induces an immunosuppressive TME^17^-^19^. Conversely, an immunosuppressive TME can induce EMT in tumor cells, creating a feedback loop between EMT and immunosuppressive conditions that promotes tumor development^20^. Several researchers have constructed EMT-related risk models to predict the prognosis of gastric cancer patients and the level of immune cell infiltration^21^, ^22^.

Recent studies suggest that there is a reciprocal relationship between AR and EMT, with EMT promoting AR and vice versa, thereby creating a feedback loop that promotes tumor progression^10^, ^23^-^25^. EMT contributes to AR by enabling cancer cells to adapt to detachment through changes in cell adhesion, cytoskeletal dynamics, microenvironmental interactions, apoptosis regulation, and metabolic adaptations^26^-^28^. AR enables cancer cells to evade apoptosis during detachment, facilitating their transition to a more invasive, metastatic phenotype^10^. Recent studies have utilized gene signatures related to EMT or anoikis to evaluate the outcomes of patients with tumors and predict immunotherapy response^29^, ^30^. However, their combined impact on prognosis and response to immunotherapy in gastric cancer has not been thoroughly investigated. Therefore, it is necessary to analyze anoikis-related and EMT-related genes (AERGs) collectively to more accurately identify the molecular subtypes of patients with gastric cancer, predict patient outcomes, and guide treatment decisions.

This study comprehensively analyzed bulk mRNA data from multiple datasets. Initially, we screened 354 AERGs and conducted prognostic and gene mutation analyses. We subsequently utilized the expression levels of AERGs to classify individuals with gastric cancer into clusters A and B. Next, we developed and validated an AERG-related model capable of predicting the outcomes of gastric cancer patients, distinguishing their TME, and predicting the effectiveness of immunotherapy and sensitivity to anticancer drugs. Finally, we further confirmed the expression of the 8 key genes in the AERG-related model through reverse transcription quantitative real-time polymerase chain reaction (RT‒qPCR) and immunohistochemical (IHC) staining analysis.

## Materials and methods

### Data gathering and preliminary processing

In this study, we downloaded the transcriptome data, tumor mutation data, and clinicopathological characteristics and survival data of gastric cancer patients from the Genomic Data Commons (GDC) database (https://portal.gdc.cancer.gov/) for The Cancer Genome Atlas of Stomach Adenocarcinoma (TCGA-STAD) dataset. The TCGA-STAD dataset comprises 32 gastric normal tissue samples and 375 gastric cancer tissue samples. Transcriptome data and corresponding clinical information from the GSE84437 and GSE62254 datasets were obtained from the Gene Expression Omnibus (GEO) database (https://www.ncbi.nlm.nih.gov/geo/). Patients with short-term survival may not have received complete treatment due to acute conditions or other reasons, which could affect the results of survival analysis. Samples with missing survival status data and survival times less than 30 days were excluded, leading to a total of 1104 gastric cancer samples included in this study, comprising 371 TCGA-STAD samples, 433 GSE84437 samples and 300 GSE62254 samples. To establish a robust risk model with a larger sample size, 804 samples from the TCGA-STAD dataset and the GSE84437 dataset were utilized to construct the risk model. The GSE62254 dataset served as an external validation cohort for evaluating the performance of the risk model. To address batch effects between different databases, the fragments per kilobase million (FPKM) format data from the TCGA-STAD dataset were converted to transcripts per kilobase million (TPM) format data and merged with the GSE84437 dataset. Then, the ComBat algorithm from the R package “sva” was employed to correct for batch effects using empirical Bayes adjustments to remove unwanted variation associated with batch labels while preserving biological variability^7^. The ComBat method adjusts gene expression values using the following transformation:



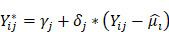




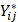
 is the batch-corrected expression of gene 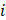 in sample 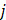, 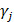 and 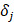 are batch-specific location and scale parameters, and 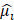 is the estimated overall mean of gene 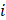. Post-correction, we performed principal component analysis (PCA) on the corrected dataset to assess whether the samples from different batches clustered together more closely and compared the distribution of expression values before and after correction using box plots to ensure that batch-related discrepancies had been minimized. The 1184 EMT-related genes were sourced from dbEMT 2.0^31^ (http://dbemt.bioinfo-minzhao.org/) (**Table S1**). As there is currently no specialized database for anoikis, we aimed to gather a comprehensive set of anoikis-related genes from multiple reliable sources. Specifically, we obtained 137 anoikis-related genes from the Harmonizome 3.0 dataset (https://maayanlab.cloud/Harmonizome/), 912 genes from the GeneCards database (https://www.genecards.org/), and 280 genes from the NCBI gene database (https://www.ncbi.nlm.nih.gov/gene). After merging and removing duplicates, we identified 916 anoikis-related genes for our study (**Table S2**).

### Screening and mutation profiling of differentially expressed prognosis-related AERGs

In this study, AERGs were derived from the intersection of anoikis-related genes and EMT-related genes. Using the TCGA-STAD dataset, a differential expression analysis of AERGs was conducted between gastric cancer tissues and adjacent normal tissues using R package “limma” with the criteria of | log_2_ fold change (FC) | >1 and adjusted *P* < 0.05^7^. The differentially expressed AERGs, together with patient survival status and survival time, were subjected to univariate Cox regression analysis. Additionally, Spearman correlation analysis was performed on the basis of the mRNA expression levels of these genes. The “maftools” package was used to create a mutation-based waterfall plot of the AERGs^32^. Finally, the prevalence of copy number variations (CNVs) in these genes and their chromosomal locations were assessed.

### Consensus unsupervised clustering analysis of AERGs

In this work, 37 AERGs were subjected to consensus unsupervised clustering analysis with k-values ranging from 2 to 9 using the R package “ConsensusClusterPlus”^33^. PCA, uniform manifold approximation and projection (UMAP), and t-distributed stochastic neighbor embedding (t-SNE) were employed as unsupervised dimensionality reduction algorithms to uncover structures in the high-dimensional data^34^-^36^. Additionally, the differences in the expression of AERGs between the two clusters were examined. Kaplan‒Meier survival analysis and heatmaps were used to investigate variations in clinical characteristics among different molecular clusters. The variances in biological functions between the two clusters were subsequently explored.

### Construction and validation of an AERG-related model

To construct a robust risk model with a larger sample size, a total of 804 gastric cancer samples (the entire cohort) from the TCGA-STAD dataset and GSE84437 dataset were divided into a discovering cohort and a testing cohort at a ratio of 7:3^7^, ^37^. The discovering cohort was used for model construction, while the testing cohort and the entire cohort were considered internal validation cohorts. The GSE62254 dataset was used as the external validation cohort. In the discovering cohort, 37 prognosis-related AERGs were subjected to least absolute shrinkage and selection operator (LASSO) Cox regression analysis to reduce overfitting among genes. The optimal λ value was determined using the R package “glmnet” via default parameters: nlambda=100, alpha=1, nfolds=10^38^. Multivariate Cox regression analysis was subsequently performed to identify the gene signature and regression coefficients of the AERG-related model. The risk score for each patient was calculated based on the gene expression and the coefficients obtained from the multivariate Cox regression model^7^, ^8^. The formula for the risk score is:



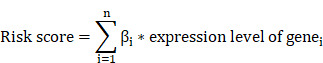



Patients with gastric cancer were divided into high-risk and low-risk categories on the basis of the median value of the risk score. In the two internal validation cohorts and one external validation cohort, a risk score was computed for each gastric cancer patient using the regression coefficients obtained from the discovering cohort. Furthermore, Kaplan‒Meier survival analysis was conducted between high- and low-risk categories via the R packages “survminer” and “survival” alongside patient survival time and status^7^, ^29^. The R package “timeROC” was utilized to generate 1-year, 3-year, and 5-year receiver operating characteristic (ROC) curves for each cohort of gastric cancer patients^7^.

### Survival analysis of subgroups with clinicopathologic features

To further confirm the performance effectiveness of the AERG-related model, we initially conducted a risk score comparison between age, sex, T stage, and N stage. We subsequently conducted survival difference analyses among different subgroups on the basis of age (≤ 65 years or > 65 years), sex (female or male), T stage (T1-2 or T3-4), and N stage (N0 or N1-3).

### Prognostic analysis and nomogram construction

This research integrated existing clinicopathological characteristic features and risk scores and conducted univariate and multivariate Cox regression analyses to further investigate the impact of the AERG-related model on prognosis. To predict the 1-, 3-, and 5-year survival rates of gastric cancer patients more accurately, the nomogram was developed by combining clinicopathological characterization factors and risk scores using the R package “rms”^8^. Calibration curves, cumulative risk curves and multivariate ROC curves were used to validate the accuracy of the nomogram.

### Analysis of the tumor immune microenvironment

To explore the role of the risk score in predicting the degree of a hot and cold TME in gastric cancer patients, we initially analyzed the levels of stromal cell and immune cell infiltration in the TME between high- and low-risk categories using the ESTIMATE algorithm^39^. We subsequently examined the Spearman correlation between the risk score and immune cell infiltration levels using 7 algorithms (XCELL, TIMER, QUANTISEQ, MCPCOUNTER, EPIC, CIBERSORT-ABS, and CIBERSORT)^7^, ^8^. To further investigate the correlation between the expression levels of the 8 model genes and immune cell infiltration levels, we analyzed the Spearman correlation among 20 immune cells and the 8 core genes based on the CIBERSORT algorithm^40^. Additionally, we assessed differences in the level of immune cell infiltration in the high- and low-risk categories using the CIBERSORT algorithm^40^. The gene expression data were input in the TPM format. The LM22 signature matrix, which is pre-built in CIBERSORT (accessed on August 2023), was used to deconvolute 22 immune cell types. We ran the analysis with 1000 permutations to ensure robust and reliable results. The default settings were used for quantile normalization as suggested for RNA-seq data.

### Immunotherapy response and antitumor drug sensitivity analyses

The tumor mutational burden (TMB), microsatellite instability (MSI), immunophenoscore (IPS), and tumor immune dysfunction and exclusion (TIDE) score were utilized to predict the efficacy of immunotherapy^7^, ^41^. Initially, we analyzed the cascade of gene mutations in the high- and low-risk categories. We investigated the correlation between the risk score and TMB using Spearman's method and explored the differences in TMB between the high- and low-risk categories. For survival analysis, we integrated the risk status and TMB status of the patients. Additionally, we obtained MSI data and the IPS of gastric cancer patients from The Cancer Immunome Atlas (TCIA) database (https://www.tcia.at/) and analyzed the differences between the high- and low-risk categories. Furthermore, we obtained TIDE scores from the TIDE database (http://tide.dfci.harvard.edu/) and evaluated the differences between the high- and low-risk categories.

Next, we performed drug sensitivity analysis for gastric cancer samples in high- and low-risk groups using gene expression and drug response data from the Genomics of Drug Sensitivity in Cancer (GDSC) database (https://www.cancerrxgene.org/). The gene expression data of gastric cancer samples were integrated with the expression profiles from the GDSC database, and batch effects were corrected using empirical Bayesian methods^42^. Drug sensitivity predictions were conducted using the calcPhenotype function from the oncoPredict package, leveraging GDSC expression data as the training dataset^43^. Sensitivity scores for multiple drugs were calculated for each gastric cancer sample. Finally, differences in drug sensitivity between high- and low-risk gastric cancer groups were assessed.

### Validation of gene expression levels and protein levels in the AERG-related model

Differential expression analysis of core genes in normal and gastric cancer tissues was conducted using the TCGA-STAD dataset and the Genotype-Tissue Expression (GTEx) dataset from the Gene Expression Profiling Interactive Analysis database (GEPIA) (http://gepia.cancer-pku.cn/). The analysis employed a cutoff criterion of | log_2_ FC | > 0.585 and *P* < 0.05. The Human Protein Atlas (HPA) database (https://www.proteinatlas.org/) provides protein and RNA profiles of human tissues and cells. We downloaded IHC staining images of the CD24, CRYAB, MMP11, MUC4, PRKAA2, SERPINE1, SKP2, and TP53 proteins in gastric normal and gastric cancer tissue samples from HPA database^44^.

### Cell lines, cell culture, and RT‒qPCR

The human stomach mucosal epithelial cell line GES-1 was acquired from Shanghai Fuheng Biologicals, while the gastric cancer cell lines AGS and HGC-27 were obtained from the cell bank of the Chinese Academy of Sciences. Cells were cultured in a humidified incubator at 37 °C with 5% CO_2_. GES-1 cells were cultured in Dulbecco's modified Eagle's medium (DMEM, Sangon Biotech, China) supplemented with 10% fetal bovine serum (FBS, Gibco, USA) and 1% penicillin-streptomycin (Beyotime, China). AGS cells were cultured in Ham's F-12 medium (Pricella, China) supplemented with 10% FBS and 1% penicillin-streptomycin. HGC-27 cells were cultured in RPMI 1640 medium (Sangon Biotech, China) supplemented with 10% FBS and 1% penicillin-streptomycin. Additional details regarding cell culture can be found in our published work^45^.

Total RNA was extracted from GES-1, AGS, and HGC-27 cells using TRIzol reagent (Invitrogen™, USA) following the manufacturer's protocol. Complementary DNA (cDNA) was synthesized using a reverse transcription kit (Takara Bio, Japan) according to the manufacturer's instructions. Approximately 1 µg of total RNA was used for each reverse transcription reaction. Random hexamers and oligo(dT) primers were included to ensure the generation of cDNA. qPCR reactions were performed using SYBR Green PCR Mix (Sangon Biotech, China) in a 96-well plate format, with a final reaction volume of 20 µL. The reactions were run on a QuantStudio 6 Flex Real-Time PCR System (Thermo Fisher Scientific) under the following cycling conditions: 95 °C for 5 minutes (initial denaturation), 40 cycles of 95 °C for 15 seconds (denaturation), 60 °C for 30 seconds (annealing), and 72 °C for 30 seconds (extension). The relative gene expression levels were calculated using the 2^(-ΔΔCt) method, normalizing the expression levels of the target genes to GAPDH. Additional details for RT‒qPCR are described in our published paper^45^. The gene primer sequences are provided in **Table S3**. All experiments were performed in triplicates to ensure data reliability.

### Statistical Analysis

Statistical analyses were performed using R software (version 4.3.0). To examine differences across categories, the Wilcoxon test was employed. OS was compared among various categories using Kaplan‒Meier curves. Univariate and multivariate Cox regression analyses were conducted to investigate independent prognostic variables. The predictive performance of the AERG-related model was assessed using ROC curves and nomograms. *P* < 0.05 was considered statistically significant. Statistical significance levels are denoted as * P < 0.05, ** P < 0.01, and *** P < 0.001.

## Results

### Prognostic value and genetic mutational landscape of AERGs

**Figure 1:**
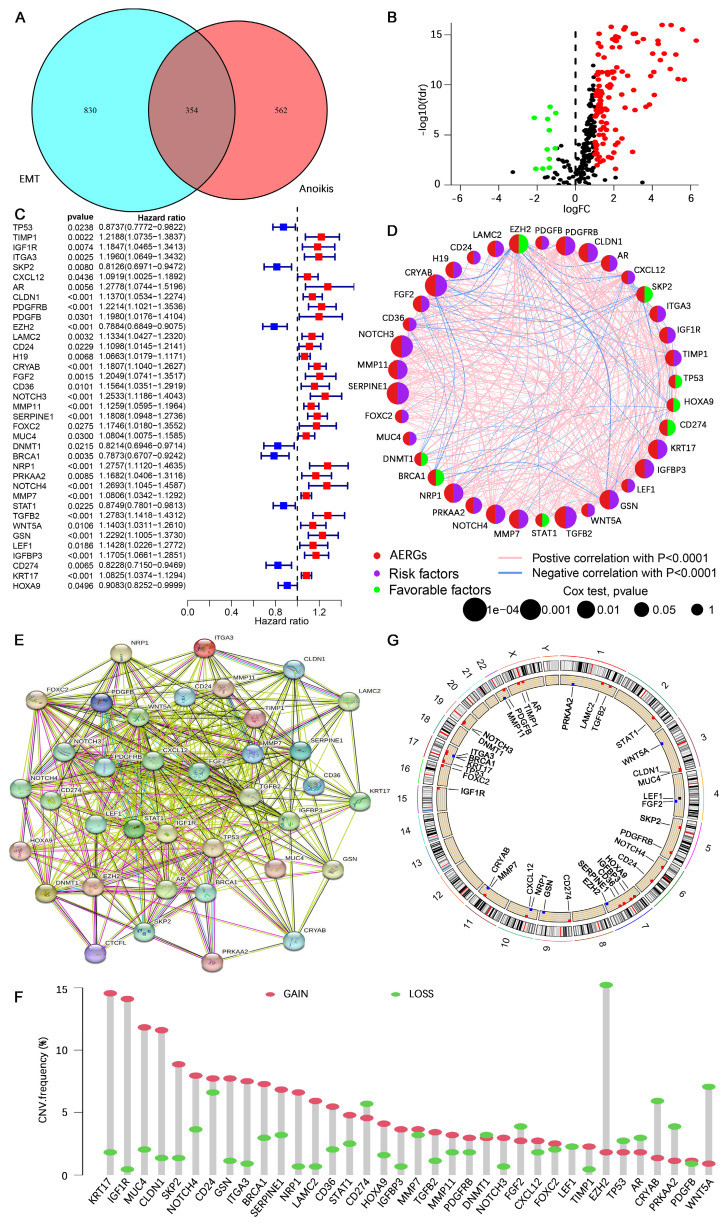
** Prognostic analysis and genetic mutation characteristics of AERGs in gastric cancer. (A)** Venn analysis of anoikis-related genes and EMT-related genes. **(B)** Differential expression analysis of 354 AERGs from the TCGA-STAD dataset. **(C)** Univariate Cox regression analysis of differentially expressed AERGs. **(D)** Correlation network analysis of 37 prognostic-related AERGs.** (E)** Protein‒protein interaction network analysis of 37 AERGs in the STRING database.** (F)** CNV frequency of gain and loss in each AERG.** (G)** CNV localization of AERGs on chromosomes. AERGs, anoikis- and EMT-related genes; EMT, Epithelial‒mesenchymal transition; TCGA-STAD, The Cancer Genome Atlas of Stomach Adenocarcinoma; CNV, copy number variation.

The study flowchart is presented in **Figure S1**. In this study, 916 anoikis-related genes and 1,184 EMT-related genes were initially subjected to Venn analysis, resulting in 354 AERGs (**Figure 1A**). The expression of these 354 AERGs was subsequently analyzed in the TCGA-STAD dataset, and 131 differentially expressed genes (DEGs) were identified. Among these DEGs, 119 genes were highly expressed in gastric cancer tissues, whereas 12 genes were expressed at low levels (**Figure 1B, and Table S4**). To examine the prognostic significance of these differentially expressed AERGs, a univariate Cox regression analysis was performed, resulting in the identification of 37 prognostically relevant AERGs. Among these genes, TP53, SKP2, EZH2, DNMT1, BRCA1, STAT1, CD274, and HOXA9 presented risk ratios less than 1, whereas the remaining 29 genes presented risk ratios greater than 1 (**Figure 1C, D and Table S5**). Additionally, protein-protein interaction (PPI) analysis revealed strong associations among the 37 proteins (**Figure 1E**). The gene mutation landscape of these 37 AERGs was explored, and the waterfall plot indicated that gene mutations were present in 63.74% of the gastric cancer patients. The genes with the highest mutation frequencies included TP53, NOTCH4, MUC4, and AR (**Figure S2**). Furthermore, gene CNV analysis revealed that KRT17, IGF1R, and MUC4 presented the greatest increase in copy number, whereas EZH2, WNT5A, and CRYAB presented the highest frequency of copy number deletion (**Figure 1F**). The distribution of the AERGs on the chromosomes is displayed in **Figure 1G**, where red dots represent genes predominantly affected by copy number increases, and blue dots represent genes predominantly affected by copy number deletions.

### Identification of two different molecular clusters based on AERGs

**Figure 2:**
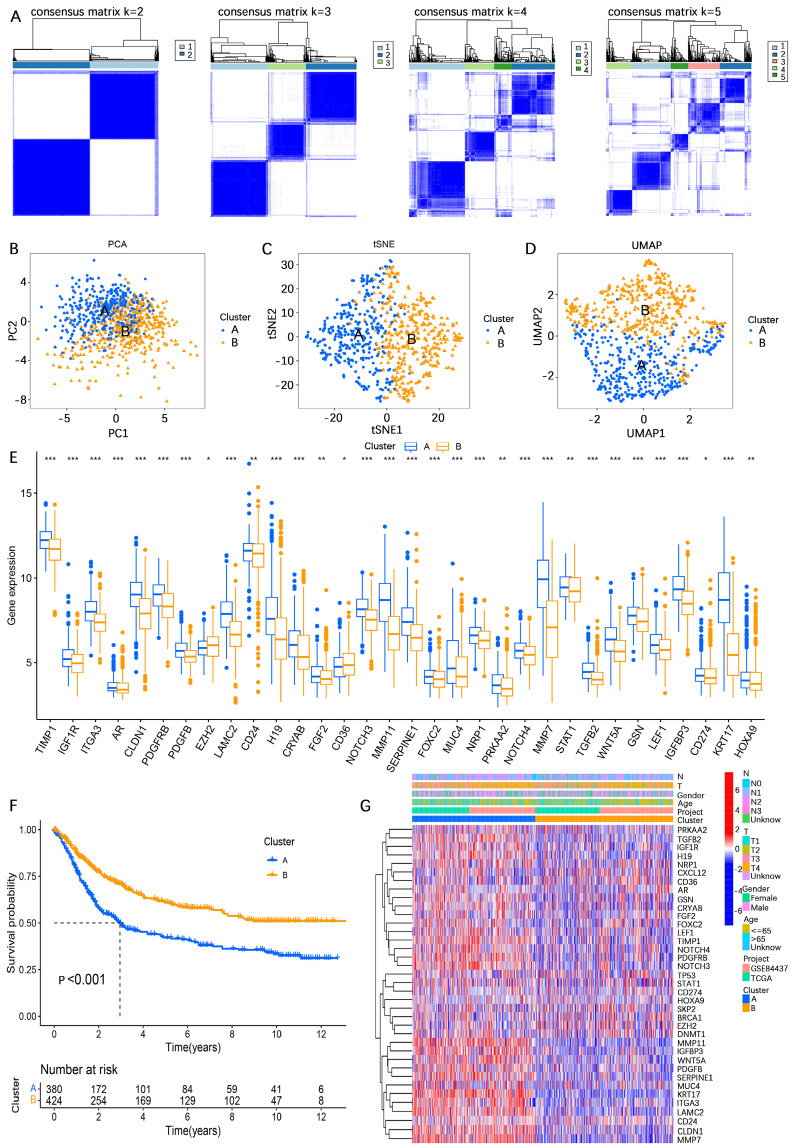
** Unsupervised clustering analysis based on the expression levels of 37 AERGs in gastric cancer. (A)** Consensus matrix heatmaps of 37 AERGs (*k* = 2-5). **(B-D)** PCA, t-SNE, and UMAP analysis of the expression profiles of different patterns. **(E)** Differences in the expression of 37 AERGs between cluster A and cluster B. **(F)** Kaplan‒Meier survival analysis between cluster A and cluster B. **(G)** Heatmap of clinicopathologic characteristics and expression levels of 37 AERGs between two different clusters. AERGs, anoikis- and EMT-related genes; PCA, principal component analysis; t-SNE, t-distributed stochastic neighbor embedding; UMAP, uniform manifold approximation and projection. *, *P* < 0.05; **, *P* < 0.01; ***, *P* < 0.001.

To comprehensively investigate the influence of the expression profiles of AERGs on prognosis and potential biological functions, unsupervised clustering analysis was performed. The results indicated that the best clustering occurred when k=2 (**Figure 2A, Figure S3,** and **Table S6**). The findings of three different downscaled clustering analyses, including PCA, t-SNE, and UMAP, further confirmed the robustness of the clustering results with k=2 (**Figure 2B-D**). A differential expression analysis of AERGs between clusters A and B revealed that the majority of genes exhibited high expression levels in cluster A, except for CD36, which was found to be expressed at low levels in cluster A (**Figure 2E**). Survival analysis demonstrated that patients belonging to cluster A had poorer overall survival (OS) than those in cluster B (**Figure 2F**). **Figure 2G** presents the clinicopathological characteristics of the patients and a heatmap depicting the expression patterns of the AERGs.

**Figure 3:**
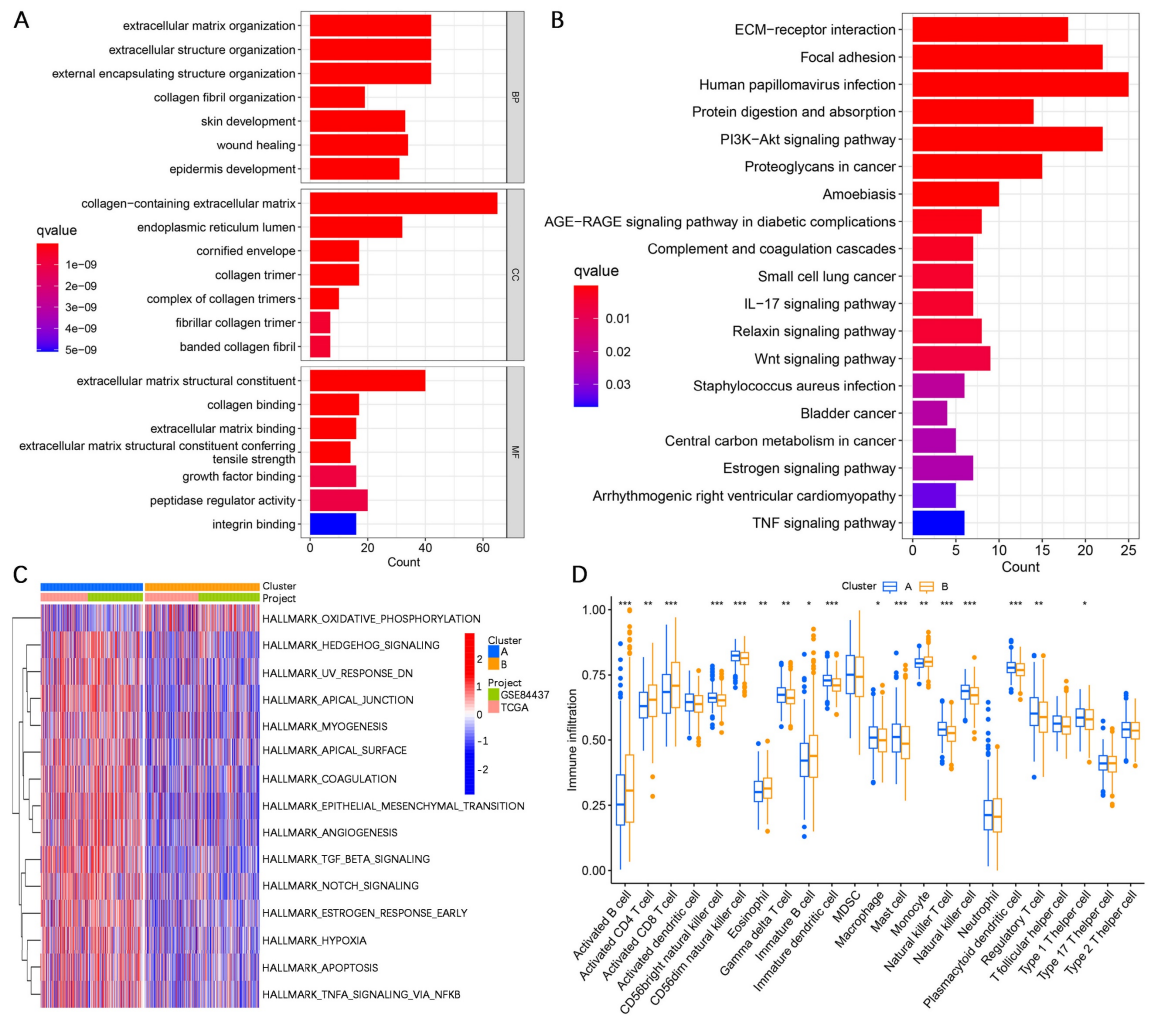
** Analysis of differences in biological functions between subgroups. (A, B)** GO functional enrichment and KEGG signaling pathway analyses of DEGs between cluster A and cluster B.** (C)** GSVA of biological pathways between clusters A and B. **(D)** Analysis of the difference in the abundance of infiltrating 23 immune cells between the two subgroups. GO, gene ontology; BP, biological process; CC, cellular components; MF, molecular function; KEGG, Kyoto Encyclopedia of Genes and Genomes. *, *P* < 0.05; **, *P* < 0.01; ***, *P* < 0.001.

To further investigate the potential biological functional impact between clusters A and B on the basis of the AERGs, we first performed differential gene expression analysis between clusters A and B and obtained 241 DEGs (**Table S7**). The 241 DEGs were then subjected to Gene Ontology (GO) functional enrichment and Kyoto Encyclopedia of Genes and Genomes (KEGG) signaling pathway analyses. GO analysis revealed that these genes were associated mostly with ECM-related functions (**Figure 3A**), whereas KEGG analysis revealed that they were associated primarily with ECM-receptor interactions and focal adhesion (**Figure 3B**). Gene set variation analysis (GSVA) analysis revealed that EMT, apoptosis, and TGF-β signaling were more active in cluster A, whereas oxidative phosphorylation was more active in cluster** B (Figure 3C**). Additionally, we examined the infiltration abundance of 23 immune cells between clusters A and B, which revealed that activated B cells, activated CD4^+^ T cells, activated CD8^+^ T cells, eosinophil, immature B cells, and monocytes had lower infiltration levels in cluster A, while CD56 bright natural killer cells, CD56 dim natural killer cells, gamma delta T cells, immature dendritic cells, macrophages, mast cells, natural killer cells, plasmacytoid dendritic cells, regulatory T cells, and type 1 T helper cells had lower infiltration levels in cluster B, whereas there was no significant difference in the infiltration abundance of activated dendritic cells, myeloid-derived suppressor cells (MDSCs), neutrophils, T follicular helper cells, type 17 T helper cells, and type 2 T helper cells between clusters A and B (**Figure 3D**).

### Construction and validation of the AERG-related model

**Figure 4:**
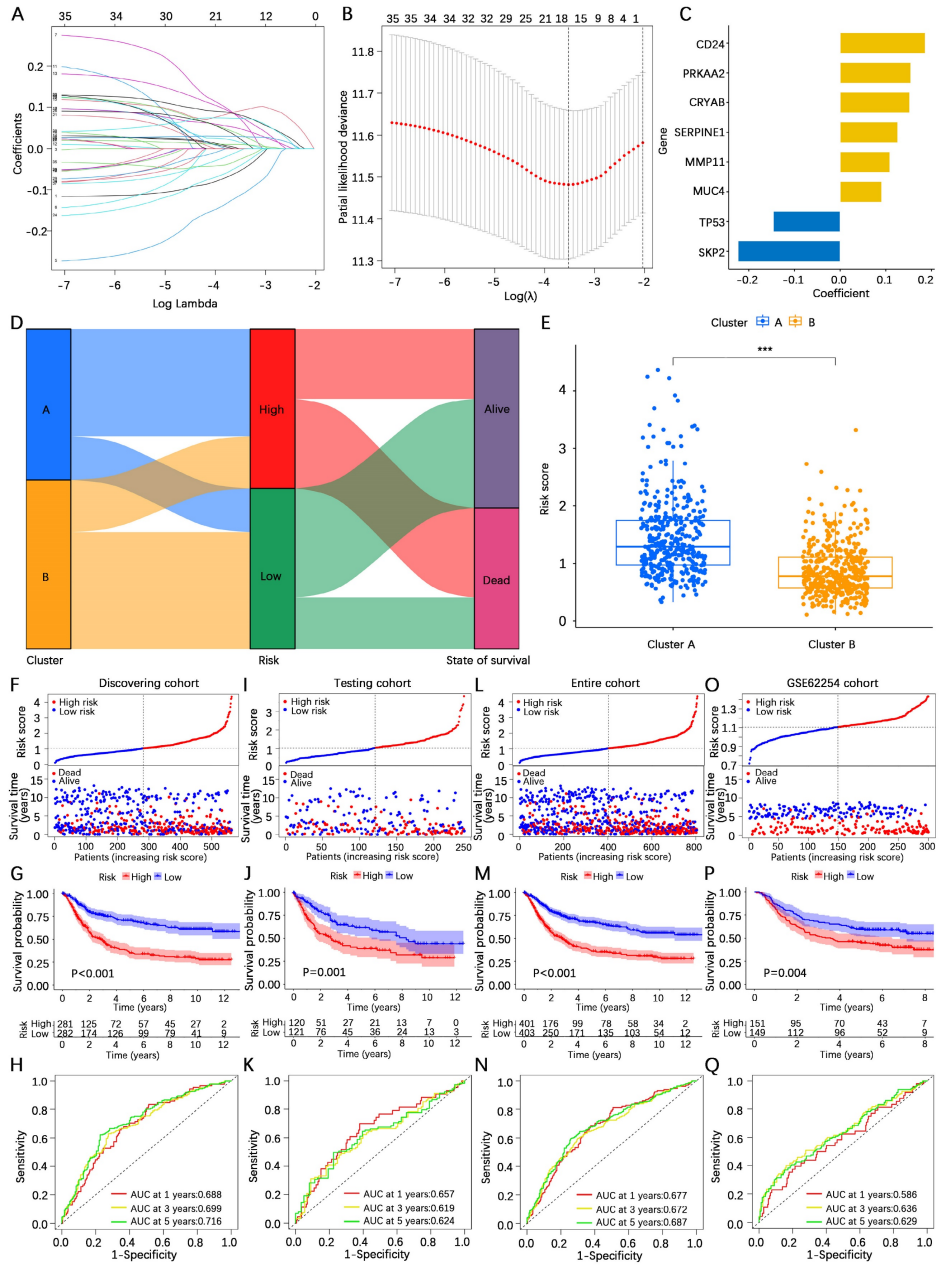
** Construction and validation of the AERG-related risk model. (A)** Dynamic process diagram for LASSO regression analysis of filtered variables.** (B)** LASSO regression analysis of cross-validated data to determine the point of minimum error. **(C)** The gene signature and regression coefficients of the AERG-related risk model were determined based on multivariate Cox regression analysis. **(D)** Sankey diagram analysis of the molecular cluster, risk level, and survival status. **(E)** Differential analysis of risk scores for molecular clusters A and B. **(F)** Patient risk score ranking chart, scatterplot of patient risk scores and survival status, **(G)** Kaplan‒Meier survival analysis between high- and low-risk categories, and **(H)** 1-, 3-, and 5-year ROC curve analysis of the discovering cohort. **(I-K)** The testing cohort, **(L-N)** entire cohort, and **(O-Q)** GSE62254 cohort were subjected to the same analysis. The testing cohort and entire cohort were internal validation cohorts, and the GSE62254 cohort was an external validation cohort. AERGs, anoikis- and EMT-related genes; ROC, receiver operating characteristic; AUC, area under the curve. ***, *P* < 0.001.

To construct an AERG-related prognostic model, we first randomized 804 gastric cancer patients (7:3) into a discovering cohort (**Table S8**) and a testing cohort (**Table S9**). In the discovering cohort, we conducted LASSO Cox regression analysis on 37 prognosis-associated genes, resulting in the identification of 16 candidate genes (**Figure 4A, B and Table S10**). The 16 candidate genes were then subjected to a multivariate Cox regression analysis, which yielded 8 core genes for building the prognostic AERG-related model (**Figure 4C**). Each gastric cancer patient's risk score was calculated, and patients were divided into high- and low-risk categories on the basis of the median risk score. The risk score for each stomach cancer patient was calculated according to the following formula: risk score = (0.1865 × expression of CD24) + (0.1524 × expression of CRYAB) + (0.1090 × expression of MMP11) + (0.0911 × expression of MUC4) + (0.1548 × expression of PRKAA2) + (0.1262 × expression of SERPINE1) + (-0.2236 × expression of SKP2) + (-0.1465 × expression of TP53) (**Table S11**). Patients were divided into high- and low-risk categories on the basis of the median risk score. Further analysis revealed that cluster A was predominantly a high-risk population, whereas cluster B was predominantly a low-risk population (**Figure 4D-E**).

To validate the performance efficacy of the AERG-related risk model, we performed validation in the discovering cohort, the two internal validation cohorts (the testing cohort and the entire cohort), and the external validation cohort (the GSE62254 cohort). In the discovering cohort, scatterplot analysis of survival status revealed a greater rate of death in patients in the high-risk categories than in patients in the low-risk categories (**Figure 4F**). Survival analysis indicated that the OS of high-risk patients was significantly lower than that of low-risk patients (**Figure 4G**). ROC curve analysis revealed that the area under the curve (AUC) values of the risk scores at 1-, 3-, and 5-year were 0.688, 0.699, and 0.716, respectively (**Figure 4H**). Furthermore, the same analyses were performed in two internal validation cohorts (**Figure 4I-N**) and the external validation cohort (**Figure 4O-Q**), which demonstrated shorter OS for gastric cancer patients in the high-risk categories.

### Subgroup expression power of the AERG-related model

**Figure 5:**
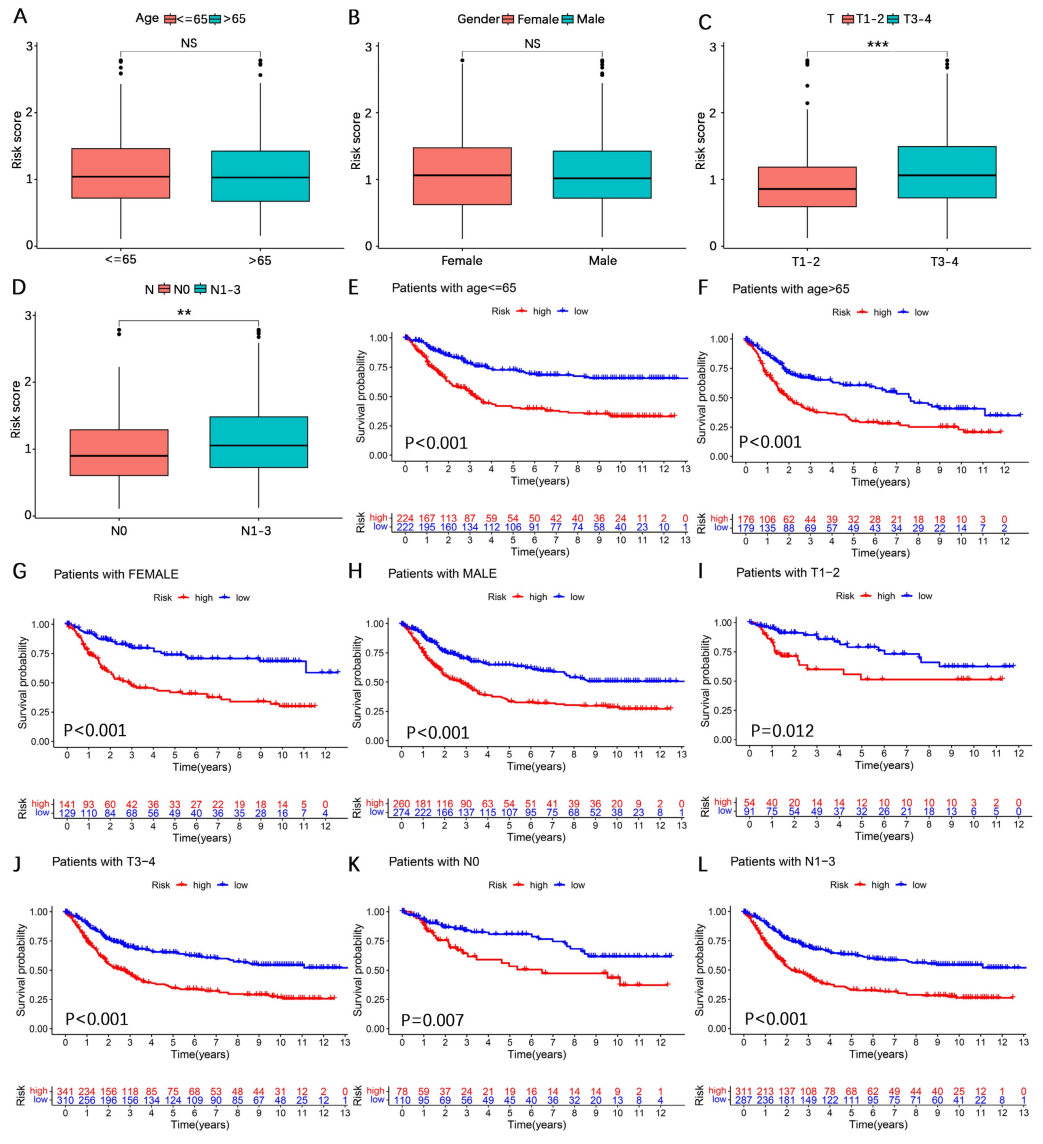
** Survival analysis between high- and low-risk categories based on clinicopathological characteristics. (A-D)** Analysis of differences in risk scores among age, sex, N stage, and T stage subgroups. **(E-L)** Survival analysis for patients aged ≤ 65 years, > 65 years, female, male, T1-2, T3-4, N0, and N1-3 between the high- and low-risk categories.

To further explore the expressive power of the AERG-related model in different clinical subgroups, we first analyzed the differences in risk scores. The findings revealed that the risk scores were higher in the T3-4 and N1-3 categories, whereas the differences in risk scores were not statistically significant according to age or sex (**Figure 5A-D**). We subsequently subdivided the clinicopathologic features into the following subgroups: ≤ 65 years of age, > 65 years of age, female, male, T1-2, T3-4, N0, and N1-3. Survival analysis revealed that, across all subgroups, high-risk patients had worse outcomes (**Figure 5E-L**).

### Prognostic analysis of the AERG-related model

**Figure 6:**
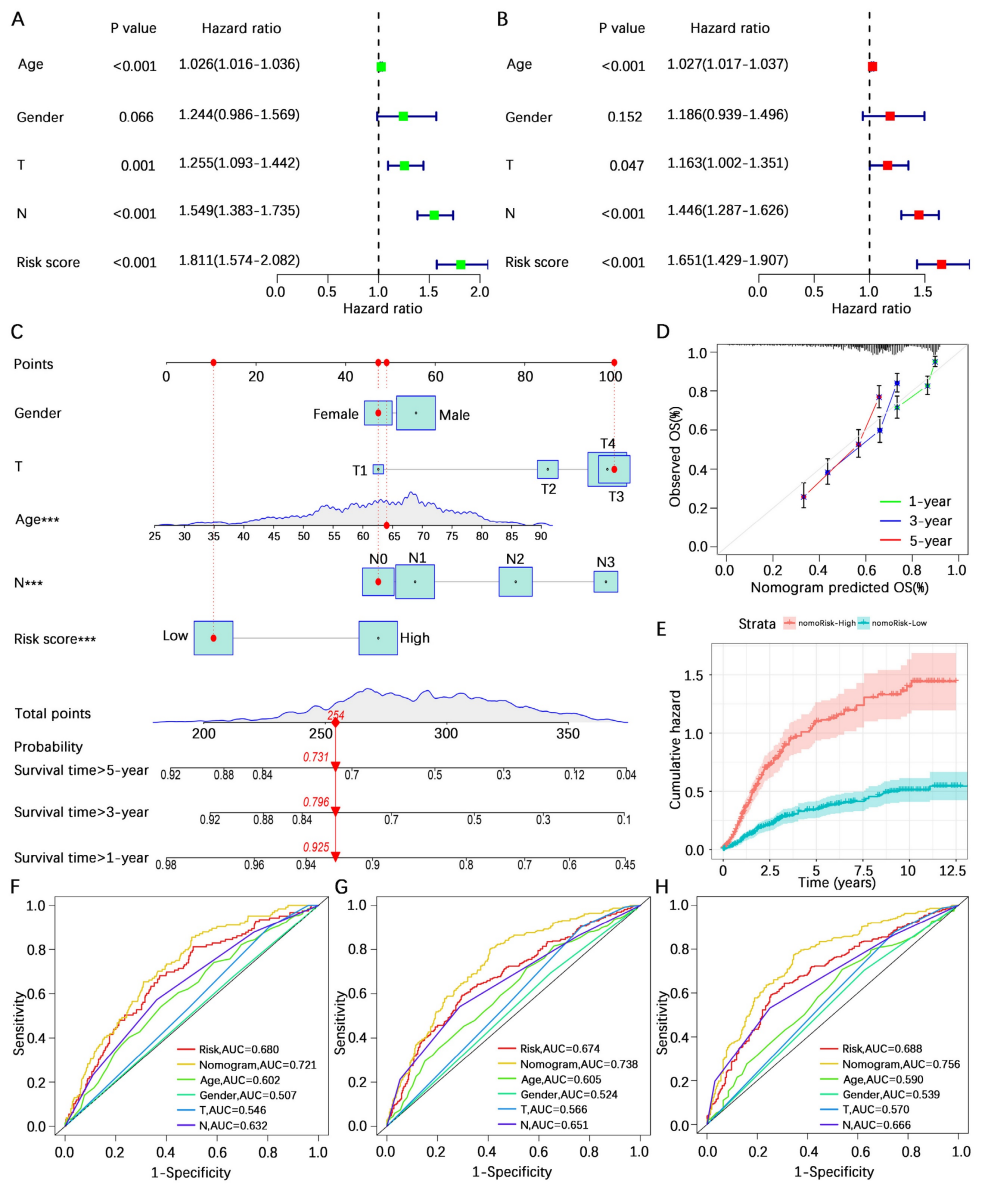
** Prognostic analysis combining the risk score and clinicopathological characteristics of patients with gastric cancer. (A, B)** Univariate and multivariate Cox regression analyses of risk scores and clinicopathological characteristics in the discovering cohort. **(C)** A nomogram was constructed based on the risk score and clinicopathological characteristics for predicting the survival rate of patients with gastric cancer at 1-, 3-, and 5-years. **(D)** Calibration plot showing the differences between the nomogram-predicted survival rates and actual survival rates. **(E)** Cumulative risk analysis of the nomogram score. **(F-H)** ROC curve analysis integrated with the nomogram score, risk score, age, sex, T stage, and N stage for patients with gastric cancer at 1-, 3-, and 5-years. AUC, area under the curve.

To further investigate the predictive value of the AERG-related model for patient prognosis, we conducted univariate and multivariate Cox regression analyses combining the risk score and clinicopathological characteristics of the patients. Univariate Cox regression analysis revealed that age (hazard ratio (HR) = 1.026, 95% confidence interval (CI) = 1.016-1.036, *P* < 0.001), T stage (HR = 1.255, 95% CI = 1.093-1.442, *P* < 0.001), N stage (HR = 1.549, 95% CI = 1.383-1.735, *P* < 0.001), and risk score (HR = 1.811, 95% CI = 1.574-2.082, *P* < 0.001) were correlated with prognosis (**Figure 6A**), and multivariate Cox regression analysis revealed that age (HR = 1.027, 95% CI = 1.017-1.037, *P* < 0.001), T stage (HR = 1.163, 95% CI = 1.002-1.351, *P* < 0.001), N stage (HR = 1.446, 95% CI = 1.287-1.626, *P* < 0.001), and risk score (HR = 1.651, 95% CI = 1.429-1.907, *P* < 0.001) were independent prognostic factors for gastric cancer patients (**Figure 6B**). In multivariate Cox regression analysis, the risk score had the highest HR value. For a more precise prediction of the 1-, 3-, and 5-year survival rates of gastric cancer patients, we constructed a nomogram by integrating clinicopathologic parameters and risk scores (**Figure 6C**), and the calibration plot illustrated that the nomogram's OS prediction results closely aligned with the actual OS outcomes (**Figure 6D**). The cumulative risk was significantly greater in patients with a high nomogram score than in those with a low nomogram score (**Figure 6E**). Additionally, the analysis of the 1-year, 3-year, and 5-year multivariate ROC curves indicated that the nomogram score had the largest AUC value (**Figure 6F-H**).

### Analysis of the tumor immune microenvironment

**Figure 7:**
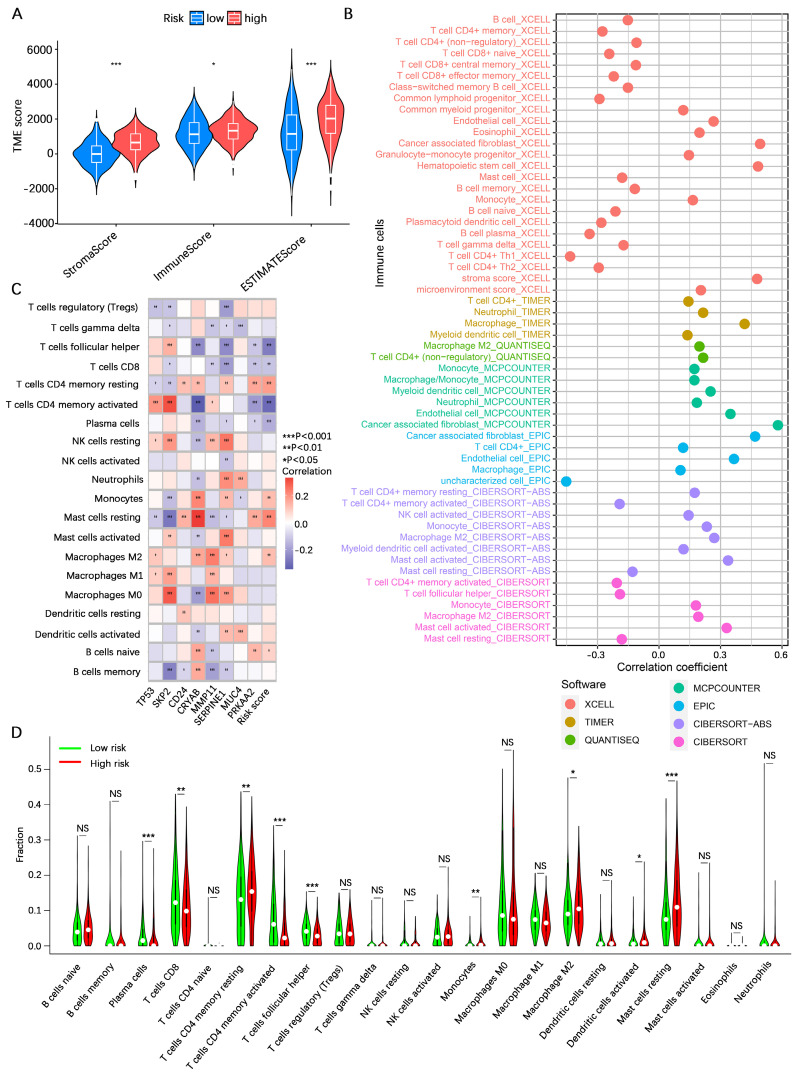
** Analysis of the tumor immune microenvironment. (A)** Analysis of differences in TME scores between high- and low-risk categories based on the ESTIMATE algorithm. **(B)** Spearman correlation analysis of risk score and abundance of immune cell infiltration using the XCELL, TIMER, QUANTISEQ, MCPCOUNTER, EPIC, CIBERSORT-ABS, and CIBERSORT algorithms. **(C)** Spearman correlation analysis of immune cell infiltration abundance and gene expression in the AERG-related model. **(D)** Analysis of differences in the abundance of 22 immune cells between high- and low-risk categories using the CIBERSORT algorithm. TME, tumor microenvironment; NS, no significance; *, *P* < 0.05; **, *P* < 0.01; ***, *P* < 0.001.

To explore the potential of the risk score in assessing the degree of hot and cold TME in gastric cancer patients, we initially analyzed the overall stromal cell and immune cell infiltration levels of patients in the high- and low-risk categories using the ESTIMATE algorithm. The results indicated that the overall stromal cell and immune cell infiltration abundances in high-risk gastric cancer patients were greater than those in low-risk gastric cancer patients (**Figure 7A**). We subsequently analyzed the correlation between the risk score and infiltration of various immune cells via seven algorithms. The results revealed positive correlations between the risk score and the levels of cancer-associated fibroblast, monocyte, macrophage M2, myeloid dendritic cell, and mast cell activated infiltration and negative correlations with the levels of B cell, mast cell, T-cell CD4^+^ Th1, and T-cell CD4^+^ Th2 infiltration (**Figure 7B**). Correlation analysis of the 8 core genes associated with the abundance of infiltrating immune cells revealed that SKP2, CRYAB, MMP11, and SERPINE1 were associated with the vast majority of immune cells, whereas TP53, CD24, MUC4, and PRKAA2 were associated with fewer immune cells (**Figure 7C**). These results suggest a correlation between the risk score and immune cell infiltration, prompting further exploration of the differences between the high- and low-risk categories. CIBERSORT analysis revealed that resting CD4^+^ memory T cells, monocytes, M2 macrophages, activated dendritic cells, and resting mast cells had greater infiltration in the high-risk category, whereas plasma cells, CD8^+^ T cells, activated memory CD4^+^ T cells, and follicular helper T cells were greater in the low-risk category (**Figure 7D**). These findings suggest that patients in the high-risk category defined by the AREG-related risk model present a more active immunosuppressive microenvironment.

### Analysis of immunotherapy response rates

**Figure 8:**
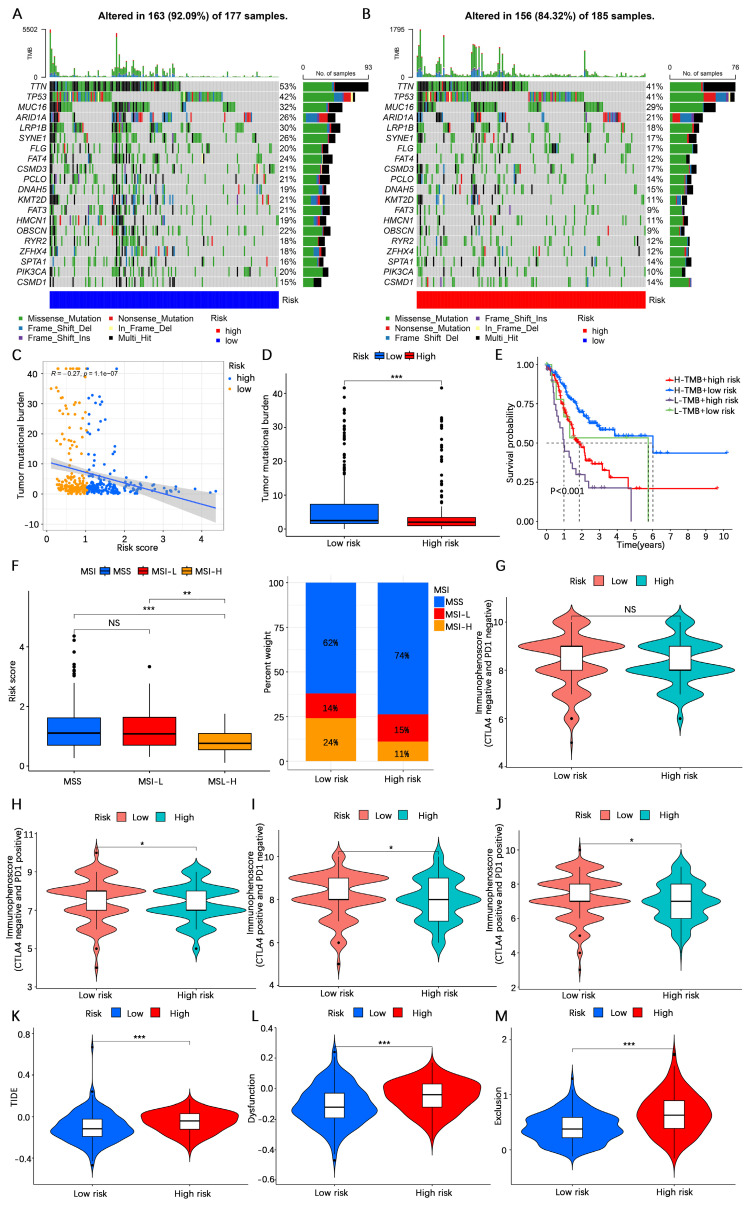
** Analysis of the immunotherapy response rate. (A, B)** Waterfall plot of tumor somatic mutations in low- and high-risk categories. **(C)** Spearman correlation analysis of the risk score and TMB. **(D)** Differential analysis of the abundance of TMB between high- and low-risk categories.** (E)** Kaplan‒Meier survival analysis among four subgroups divided by the risk score and the TMB.** (F)** Relationships between the risk score and MSI. **(G-J)** IPS score analysis between the high- and low-risk categories. **(K-M)** TIDE score analysis between the high- and low-risk categories. TMB, tumor mutation burden; MSS, microsatellite stability; MSS-L, microsatellite instability-low; MSS-H, microsatellite instability-high; IPS, immunophenoscore; TIDE, tumor immune dysfunction and exclusion. NS, no significance; *, *P* < 0.05; **, *P* < 0.01; ***, *P* < 0.001.

The TMB is considered one of the indicators of the effectiveness of cancer immunotherapy. To explore the differences in response rates to immunotherapy between the high- and low-risk categories, we first analyzed the TMB in the high- and low-risk categories based on somatic mutation profiles. The waterfall plots revealed that the proportion of somatic mutations in the low-risk category (92.09%) was greater than that in the high-risk category (84.32%) (**Figure 8A, B**). Spearman correlation analysis revealed a negative correlation between the TMB and the risk score (R= -0.27,* P* < 0.001) (**Figure 8C**). Consistent with the findings of the waterfall plot, the risk score difference analysis revealed that the TMB in the low-risk category was lower than that in the high-risk category (**Figure 8D**). In addition, we conducted a survival analysis combining the risk score and TMB, and the findings indicated that gastric cancer patients with low TMB and high-risk scores had the worst prognosis, whereas those with high TMB and low-risk scores had the best prognosis (**Figure 8E**). Studies have shown that immunotherapy is more effective in patients with high microsatellite instability (MSI-H) tumors compared to patients with low microsatellite instability (MSI-L) and microsatellite stability (MSS) tumors^46^. In our investigation, a greater prevalence of MSI-H was found in individuals at low risk (**Figure 8F**). The IPS indicates tumor tissue immunogenicity, with a higher IPS providing greater benefit to immunotherapy. Our results revealed that the IPS was greater in low-risk gastric cancer patients than in high-risk gastric cancer patients (**Figur**e **8G-J**). The TIDE score is a unique biomarker for the rate of tumor immunotherapy response; the higher the TIDE score is, the greater the risk of tumor immune escape. The TIDE score of patients in the low-risk category was lower than that of patients in the high-risk category (**Figure 8K-M**), indicating that patients in the low-risk category were less likely to experience immune escape and had a higher response rate to immunotherapy. These results suggest that patients in the low-risk category may benefit more from immunotherapy.

### Antitumor drug sensitivity analysis

**Figure 9:**
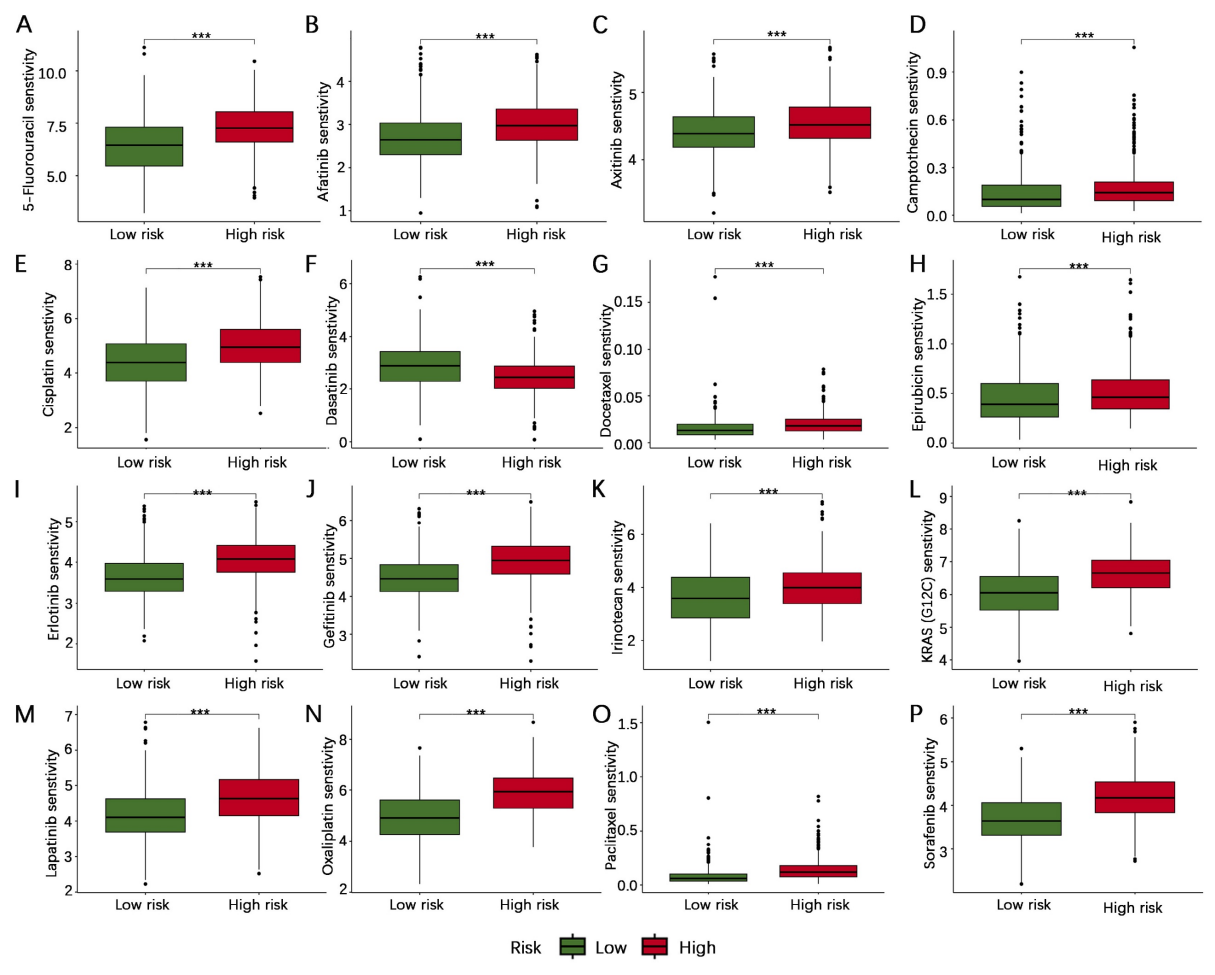
** Sensitivity analysis of antitumor drugs in patients with gastric cancer in the high- and low-risk categories. (A-P)** Sensitivity analysis of 5-fluorouracil, afatinib, axitinib, camptothecin, cisplatin, dasatinib, docetaxel, epirubicin, erlotinib, gefitinib, irinotecan, KRAS (G12C) inhibitor-12, lapatinib, oxaliplatin, paclitaxel, and sorafenib in the high- and low-risk categories. ***, *P* < 0.001.

To further investigate the possible importance of the AERG-related model in guiding the selection of anticancer medications for patients, we compared the IC50 values of drugs in high- and low-risk categories. The results indicated that 5-fluorouracil, afatinib, axitinib, camptothecin, cisplatin, docetaxel, epirubicin, erlotinib, gefitinib, irinotecan, KRAS (G12C) inhibitor-12, lapatinib, oxaliplatin, paclitaxel, and sorafenib were more sensitive in low-risk category than in the high-risk category, whereas dasatinib was less sensitive in low-risk category than in the high-risk category (**Figure 9**). These findings could guide the personalized treatment of gastric cancer patients.

### Validation of model gene expression levels

**Figure 10:**
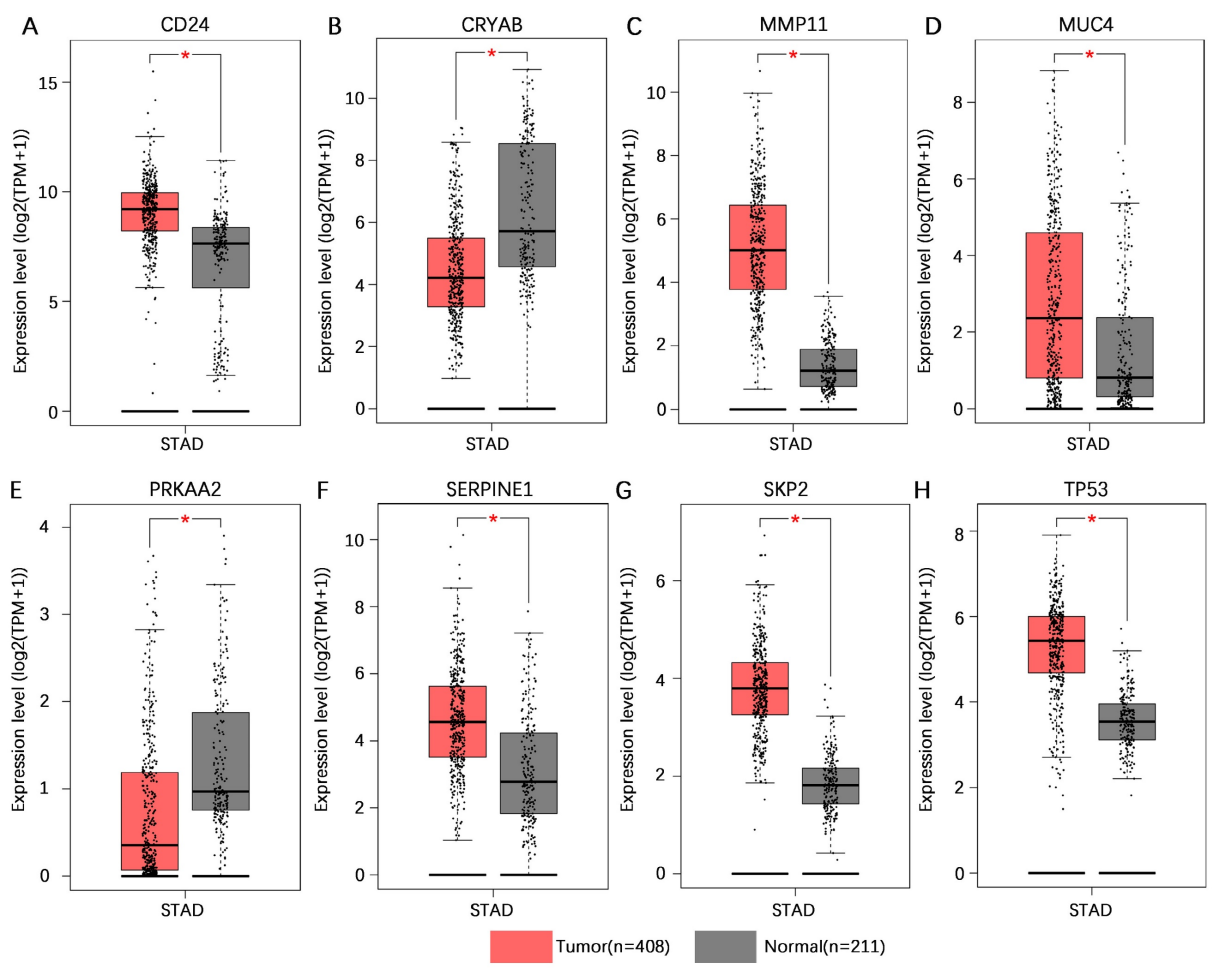
** Validation of the mRNA expression levels of 8 core genes in the AERG-related risk model in normal gastric and gastric cancer tissues. (A-H)** CD24, CRYAB, MMP11, MUC4, PRKAA2, SERPINE1, SKP2, and TP53 mRNA expression levels in normal gastric tissues and gastric cancer tissues were analyzed using the TCGA-STAD dataset and GTEx dataset in the GEPIA database. AERGs, anoikis- and EMT-related genes; TCGA-STAD, The Cancer Genome Atlas of Stomach Adenocarcinoma; GTEx, Genotype-Tissue Expression; GEPIA, Gene Expression Profiling Interactive Analysis database. *, *P* < 0.05.

To increase the number of normal gastric tissue samples, we performed joint analysis of the TCGA-STAD dataset and GTEx dataset in the GEPIA database. The findings indicated that CD24, MMP11, MUC4, SERPINE1, SKP2, and TP53 exhibited higher levels of mRNA expression in gastric cancer tissues than in normal gastric tissues, whereas CRYAB and PRKAA2 presented lower mRNA expression levels in gastric cancer tissues than in normal gastric tissues (**Figure 10**).

**Figure 11:**
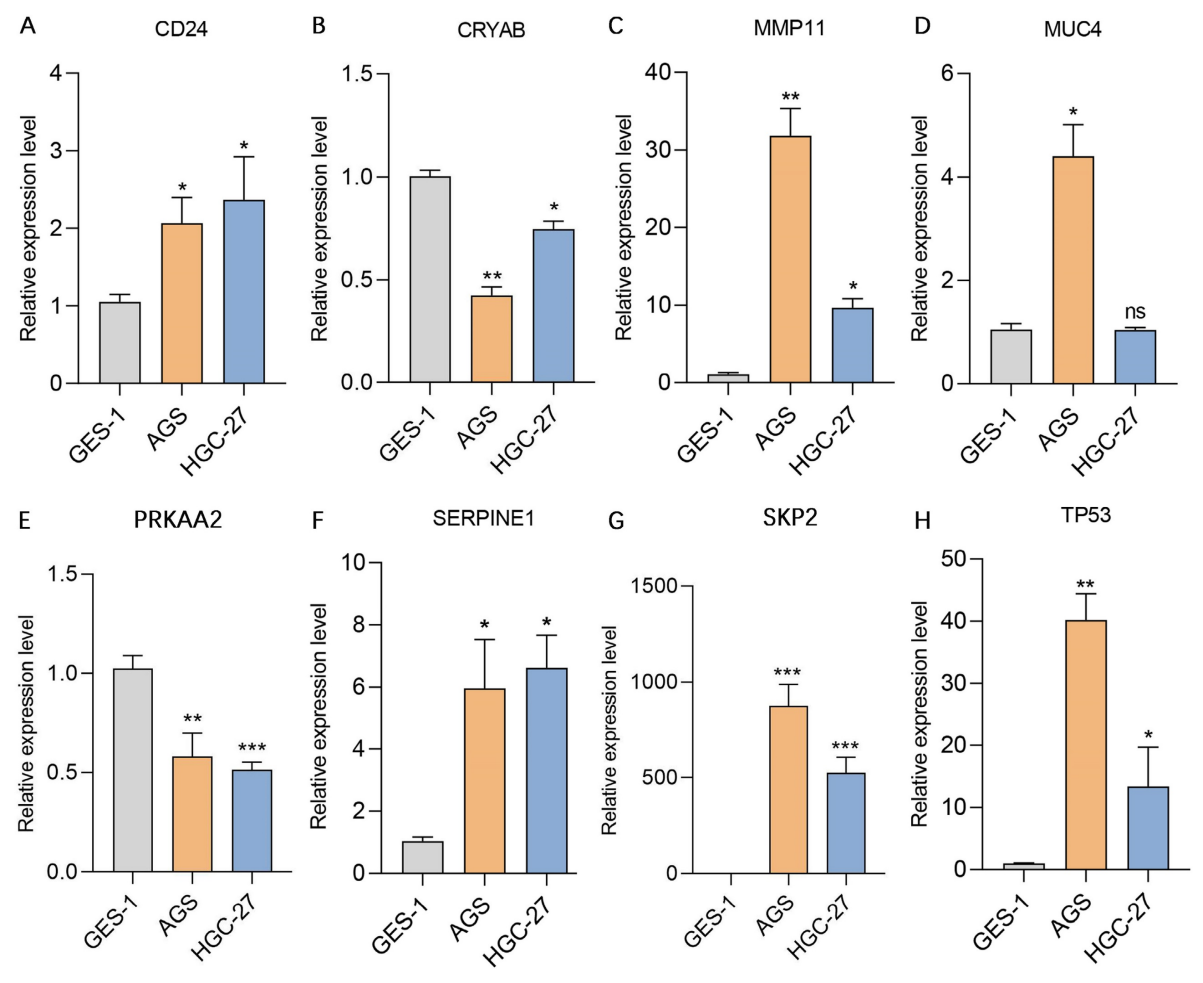
** Validation of the expression levels of 8 genes in the AERG-related model in gastric mucosal epithelial cells and gastric cancer cell lines. (A-H)** CD24, CRYAB, MMP11, MUC4, PRKAA2, SERPINE1, SKP2, and TP53 expression levels were detected in the stomach mucosal epithelial cell line GES-1 and the gastric cancer cell lines AGS and HGC-27 via RT‒qPCR. AERGs, anoikis- and EMT-related genes. *, *P* < 0.05; **, *P* < 0.01; ***, *P* < 0.001.

**Figure 12:**
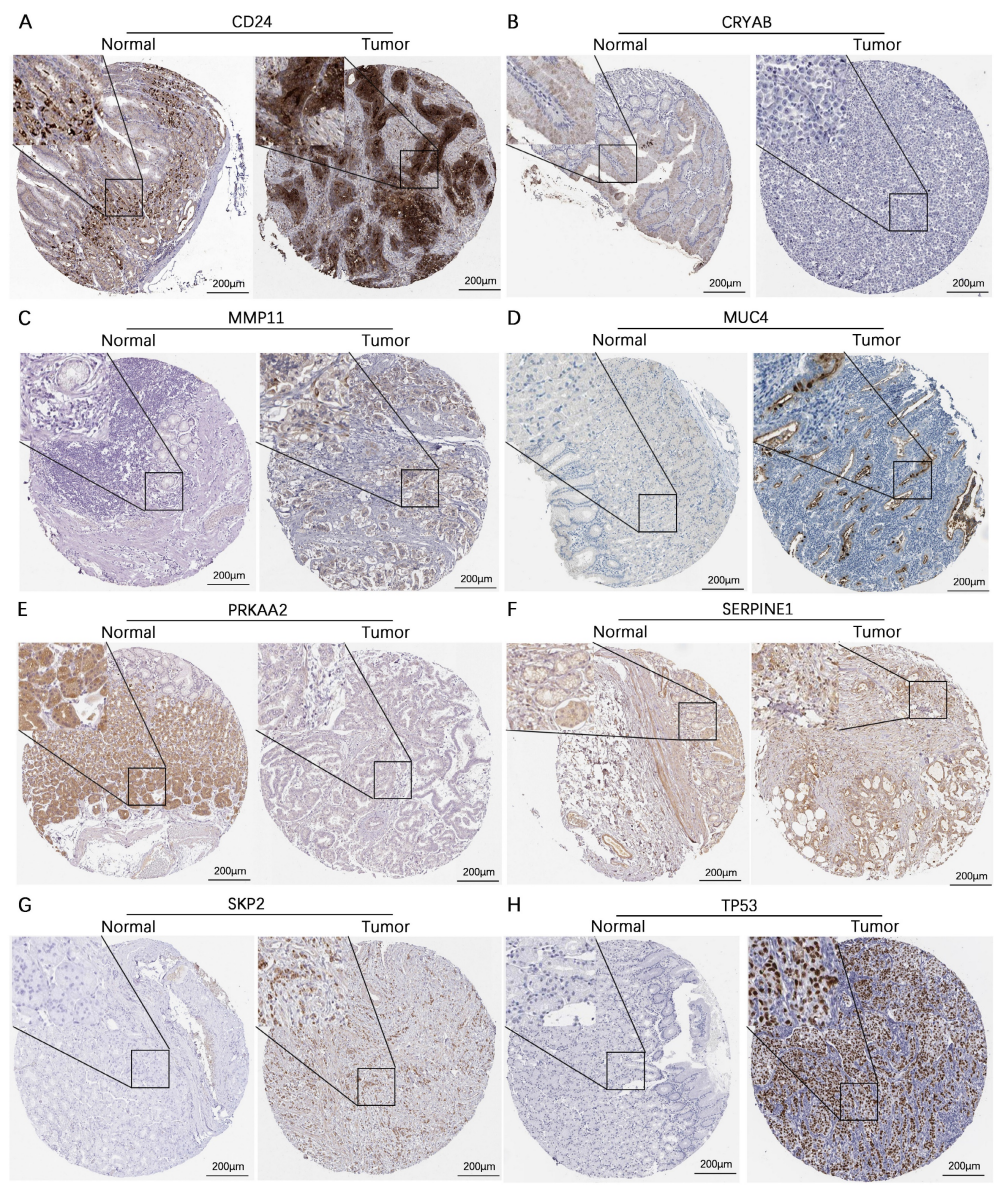
** Validation of the protein expression levels of 8 genes in the AERG-related model in normal gastric tissues and gastric cancer tissues. (A-H)** CD24, CRYAB, MMP11, MUC4, PRKAA2, SERPINE1, SKP2, and TP53 protein expression levels were analyzed in normal gastric tissues and gastric cancer tissues using the HPA database. AERGs, anoikis- and EMT-related genes. HPA, The Human Protein Atlas.

We subsequently assessed the expression levels of these 8 genes in the human gastric mucosa cell line GES-1 and the human gastric adenocarcinoma cell lines AGS and HGC-27 via RT‒qPCR. The results revealed that CD24, MMP11, SERPINE1, SKP2, and TP53 were expressed at higher levels in the AGS and HGC-27 cell lines than in the GES-1 cell line. In contrast, CRYAB and PRKAA2 were expressed at lower expression levels in the AGS and HGC-27 cell lines than in the GES-1 cell line, while MUC4 was expressed at higher expression levels in the AGS cell line than in the GES-1 cell line (**Figure 11**). Additionally, this study further analyzed the protein expression levels of model genes expressed in gastric cancer tissues and normal gastric tissues. IHC staining revealed that the protein expression levels of CD24, MMP11, MUC4, SERPINE1, SKP2, and TP53 in gastric cancer tissues were greater than those in normal gastric tissues, whereas the protein expression levels of CRYAB and PRKAA2 were lower in gastric cancer tissues than in normal gastric tissues (**Figure 12**).

## Discussion

China has the highest incidence of gastric cancer globally; however, the early diagnosis rate remains below 20%, and the prognosis for advanced gastric cancer patients is poor^1^, ^47^. Tumor metastasis is associated with the phenomenon of anchorage independence known as AR^11^. Anoikis-related genes have been utilized by researchers to develop prognostic models and assess the TME. For example, Sun *et al.* classified glioblastoma into two categories on the basis of anoikis-related genes and demonstrated that patients in category 1 had shorter survival and a more active immunosuppressive TME^29^. Aberrant activation of EMT contributes to tumor migration, invasion, and the induction of an immunosuppressive TME, leading to immune escape^20^, ^48^. Yang *et al.* developed a prognostic model for colorectal cancer patients using EMT-related genes^30^. However, most studies typically focus on only one phenotype, disregarding the fact that tumorigenesis and progression are influenced by multiple phenotypes. Studies have highlighted the interconnectedness between the two phenotypes of anoikis and EMT^10^, ^26^. Hence, it is necessary to integrate the analysis of both anoikis and EMT phenotypes to explore molecular tumor subtypes comprehensively and provide prognostic information for patients with gastric cancer, guiding individualized antitumor therapies.

In this study, a total of 354 AERGs were identified, and 37 differentially expressed AERGs associated with prognosis were discovered. Further analysis revealed that among the 37 AERGs, TP53 had the highest mutation frequency at 44%, KRT17 had the highest frequency of copy number gain, and EZH2 had the highest frequency of copy number loss. On the basis of the expression levels of these 37 AERGs, gastric cancer patients were classified into molecular cluster A and molecular cluster B using unsupervised clustering analysis. GO analysis revealed that the DEGs between the two clusters were predominantly involved in ECM-related activities. Additionally, KEGG analysis indicated that the DEGs were associated primarily with ECM-receptor interactions and focal adhesion. Similar to how soil components are essential for plant growth, the ECM provides a supportive environment for cell proliferation and survival. The clustering analysis based on the AERGs in this study revealed significant differences in the ECM between molecular clusters A and B. ECM remodeling can affect tumor proliferation, anoikis, metastasis, and immune escape^20^, further validating the reliability of the findings of this study. Further analysis demonstrated that molecular cluster A exhibited more active EMT signaling and TGF-β signaling, whereas molecular cluster B presented more active oxidative phosphorylation signaling. Moreover, the infiltration of activated B cells, activated CD4+ T cells, activated CD8^+^ T cells, and Treg cells in molecular cluster A were lower than those in molecular cluster B. Treg cells constitue a subset of CD4^+^ T cells that maintain immune tolerance by inhibiting the activity of other immune cells against one's own tissues^49^, ^50^. In tumors, Treg cells can suppress the activation and killing abilities of T cells by secreting immunosuppressive cytokines and regulating immune checkpoint molecules^51^. Aberrant activation of EMT has been correlated with an increased number of CD4^+^ Foxp3^+^ Treg cells^52^. The results of this study align with these previous reports. The hypothesis is that patients in molecular cluster A, characterized by greater Treg cell infiltration, active EMT signaling, and TGF-β signaling, may have an immunosuppressive tumor microenvironment and could be classified as "cold tumors" that potentially exhibit a worse prognosis.

Currently, tumor indicators, staging, and pathology types are frequently used to assess the prognosis of cancer patients, but the accuracy of these methods still needs improvement. Therefore, we developed an AERG-related model to more precisely evaluate the risk level and prognosis of patients. Cao *et al.* constructed a risk model related to anoikis in gastric cancer, incorporating 9 genes. The 1-year, 3-year, and 5-year AUC values of the nomogram score were 0.709, 0.717, and 0.715, respectively^15^. In this study, the risk model related to AERGs included 8 genes, and the 1-year, 3-year, and 5-year AUC values of the nomogram score were 0.721, 0.738, and 0.756, respectively. Our risk model had higher AUC values for the nomogram score while incorporating a smaller number of genes, suggesting that the effectiveness of the risk model constructed by combining multiple phenotypes is better.

The AERG-related model consists of 8 genes: CD24, CRYAB, MMP11, MUC4, PRKAA2, SERPINE1, SKP2, and TP53. CD24 is a cell-surface glycosylated protein and one of the markers for gastric cancer stem cells^53^. Wang *et al.* demonstrated that CD24 inhibits apoptosis and promotes invasion of gastric cancer cells by activating STAT3^54^. CRYAB is a small heat shock protein that can promote the migration and invasion of gastric cancer cells by mediating EMT through NF-κB signaling. It is considered a marker of a poor prognosis of gastric cancer^55^. MMP11 is significantly expressed in gastric cancer cells and has been implicated in enhancing tumor growth and invasion through the modulation of IGF-1 signaling^56^. Exosome miR-139 secreted by gastric cancer CAFs has been shown to inhibit gastric cancer progression and metastasis by reducing MMP11 in the TME^57^. MUC4, a transmembrane glycoprotein, is highly expressed in various epithelial tumors, modulates HER-2 signaling, and is considered a crucial factor in the efficacy of trastuzumab^58^. In gastric cancer, high expression of MUC4 and MUC1 is associated with poor patient prognosis^59^. PRKAA2, also known as AMPKα2, can regulate autophagy-related genes to mediate autophagy at the transcriptional level, thus promoting drug resistance in gastric cancer cells^60^. In addition, PRKAA2 can also regulate glucose metabolism and fatty acid metabolism^61^. SERPINE1, a member of the serine protease inhibitor family, plays a key role in gastric cancer by regulating cell proliferation, invasion, migration, and the EMT process^62^. Studies have shown a correlation between the expression level of SERPINE1 and immune cell infiltration, making it a potential target for immunotherapy^63^. SKP2 is regarded as an oncoprotein that is highly expressed in a variety of tumors and can regulate tumor cell proliferation, invasion and metabolism by promoting the ubiquitination of the p27 and p21 proteins^64^. TP53 is classified into wild-type and mutant types; wild-type TP53 is considered a tumor suppressor gene, whereas mutant TP53 is regarded as an oncogene^65^. Nie *et al.* constructed a TP53-associated immune prognostic model that predicted the outcomes of gastric cancer patients, and TP53 mutation downregulated the immune response in gastric cancer^66^.

The TME refers to the internal environment of the tumor and consists mainly of tumor cells, stromal cells such as CAFs, immune cells, cytokines, and ECM components^6^. In the early stages of tumor development, the TME has a tumor-suppressive role; however, as the tumor progresses, it undergoes changes that lead to immune tolerance and a shift toward a tumor-promoting microenvironment^67^. Alterations in the inhibitory aspects of the TME are crucial in influencing the effectiveness of immunotherapy. CAFs, for example, can secrete TGF-β to inhibit the maturation of DCs and promote the differentiation of Treg cells. They can also release IL-6 to promote the differentiation of MDSCs and inhibit the activity of cytotoxic T cells^68^. Additionally, CAFs can generate excessive collagen, which remodels the ECM, creating a barrier that limits drug penetration and immune cell infiltration^69^, ^70^. TAMs can aid in immune evasion by producing immunosuppressive factors such as IL-10, prostaglandin E2, and TGF-β^71^. Moreover, the interaction between the CD47 protein on tumor cells and signal regulatory protein alpha (SIRPα) on macrophages can activate a "don't eat me" signal, enabling tumor cells to evade immune surveillance and attack^72^. Treg cells contribute to immunosuppression by secreting cytokines that inhibit the activation and cytotoxicity of T cells, as well as by modulating immune checkpoint molecules^51^. This study revealed that patients in the high-risk category had higher stromal scores and immune scores than those in the low-risk category did, indicating an active TME in high-risk patients. Further analysis revealed a positive correlation between the risk score and the infiltration of MDSCs, CAFs, and TAMs, suggesting that higher-risk gastric cancer patients exhibit greater infiltration of these immunosuppressive cells. These findings indicate that these cells contribute to an immunosuppressive TME, thereby promoting immune evasion and tumor progression. Therefore, we postulate that an immunosuppressive TME may contribute to the poor prognosis observed in individuals with high-risk gastric cancer.

Immunotherapy has brought new hope to cancer patients, including those with gastric cancer. Different studies have shown varying efficacy rates of ICIs in different gastric cancer patients^73^. Currently, common markers used to predict an ICI response include the TMB, PD-L1 expression, MSI status, and circulating tumor DNA^73^. Studies have indicated that mutations leading to the production of more neoantigens enhance T-cell recognition, and ICIs reactivate T cells, making them more effective in individuals with high TMB in clinical settings^74^. However, some mutations may be ineffective or irrelevant to antigen production, so tumors with high TMB do not necessarily respond well to ICIs. MSI-H, which is caused mostly by MMR gene defects, results in the generation of neoantigens from unrepaired misreplicated DNA, leading to increased infiltration of tumor-infiltrating lymphocytes (TILs). Patients with MSI-H tumors typically have greater TMB and show a better response to ICIs than patients with MSS tumors^46^. However, the overall incidence of MSI-H tumors is about 10% in gastric cancer^75^. Therefore, as a biomarker, MSI-H has limited applicability to certain populations. The IPS is calculated on a scale of 0-10 based on the expression levels of representative genes or genomes in the immunophenogram^76^. A higher IPS indicates better immunogenicity and better efficacy for immunotherapy. The TIDE score, a unique biomarker for the tumor immunotherapy response, indicates the likelihood of tumor immune escape. Higher TIDE scores are associated with a greater risk of immune escape^7^, ^8^. In this study, a negative correlation was observed between the risk score and TMB, with low-risk patients exhibiting a higher frequency of MSI-H, higher IPS, and lower TIDE scores. These findings suggest that patients in the low-risk category are more likely to respond to ICIs, potentially benefiting from immunotherapy. Moreover, survival analysis revealed that patients in the high-TMB and low-risk category had the best prognosis, whereas those in the low-TMB and high-risk category had the worst prognosis. These findings indicate that the risk score may serve as a new marker for predicting the response rate to ICIs, potentially improving the efficacy of response rate prediction when used in combination with the TMB. Additionally, variations in the IC50 values of antitumor medications between high-risk and low-risk categories were evaluated, aiming to provide guidance for selecting appropriate clinical antitumor agents and achieving personalized diagnosis and therapy.

This study has the following strengths. First, this study combines genes related to anoikis and EMT. Both mechanisms play critical roles in tumor metastasis and immune evasion, allowing for a more comprehensive understanding of tumor progression. Second, our model assesses patient sensitivity to anticancer drugs, offering dual functionality. This helps guide decisions not only for immunotherapy but also for traditional chemotherapy, providing a practical advantage over existing models that typically focus on prognosis or immunotherapy response alone. Third, by integrating the risk score with clinicopathological features, we developed a nomogram that is easy to apply in clinical settings, making the model more practical for real-world use. This is a significant advantage over models lacking user-friendly tools for clinical implementation.

This research has certain limitations. First, the selection of anoikis- and EMT-related genes was based on predefined gene sets from previous literature and public databases. While these genes are known to be involved in tumor biology, some important genes might have been overlooked, and there could be biases associated with the initial selection of gene sets. Further exploration and validation of other potential AERG candidates would improve the model's comprehensiveness. Second, this study relies on publicly available datasets (TCGA and GEO), which may not fully represent the diversity of gastric cancer patients globally. A larger and more diverse cohort, including multi-center data and different ethnic groups, would enhance the generalizability of our findings. Third, although we applied the ComBat algorithm to correct for batch effects between the datasets, such corrections may not fully account for all sources of technical variation. This could lead to subtle biases in gene expression data. Future studies could benefit from improved harmonization techniques and the inclusion of additional datasets to validate the robustness of our model. And last but not least, the proposed AERG-related risk model is based on bioinformatics analysis. Although we conducted preliminary validation using RT‒qPCR and IHC for selected genes, further functional studies, such as *in vitro* or *in vivo* assays, are needed to confirm the biological relevance of these genes in anoikis resistance and EMT.

## Conclusion

In summary, this research developed an AREG-related model that can predict the outcomes of patients with gastric cancer, react to the condition of the TME, and predict the rate of immunotherapy response and antitumor drug sensitivity.

## Supplementary Material

Supplementary tables.

Supplementary figures.

## Abbreviations

AR: Anoikis Resistance
EMT: Epithelial‒Mesenchymal Transition
AERGs: Anoikis- and EMT-Related Genes
AUC: Area Under the Curve
BP: Biological Process
CAFs: Cancer-Associated Fibroblasts
CC: Cellular Components
CNV: Copy Number Variations
ECM: Extracellular Matrix
FC: Fold Change
FPKM: Fragments Per Kilobase Million
GDC: Genomic Data Commons
GDSC: Genomics of Drug Sensitivity in Cancer
GEO: Gene Expression Omnibus
GEPIA: Gene Expression Profiling Interactive Analysis
GO: Gene Ontology
GSVA: Gene Set Variation Analysis
GTEx: Genotype-Tissue Expression
HER2: Human Epidermal Growth Factor Receptor 2
HPA: The Human Protein Atlas
IC50: Half Maximal Inhibitory Concentration
ICIs: Immune Checkpoint Inhibitors
IPS: Immunophenoscore
KEGG: Kyoto Encyclopedia of Genes and Genomes
LASSO: Least Absolute Shrinkage and Selection Operator
MF: Molecular Function
MSI: Microsatellite Instability
MSS: Microsatellite Stability
MSS-H: Microsatellite Instability-High
MSS-L: Microsatellite Instability-Low
PCA: Principal Component Analysis
PPI: Protein-Protein Interaction
ROC: Receiver Operating Characteristic
RT‒qPCR: reverse transcription quantitative real-time polymerase chain reaction
STAD: Stomach Adenocarcinoma
TCGA: The Cancer Genome Atlas Program
TCGA-STAD: The Cancer Genome Atlas of Stomach Adenocarcinoma
TCIA: The Cancer Immunome Atlas
TGF-β: Transforming Growth Factor-beta
TIDE: Tumor Immune Dysfunction and Exclusion
TMB: Tumor Mutation Burden
TME: Tumor Microenvironment
TPM: Transcripts Per Kilobase Million
Treg: Regulatory T
t-SNE: t-Distributed Stochastic Neighbor Embedding
UMAP: Uniform Manifold Approximation and Projection
