# Supplementary material for: Training and experimental validation a novel anoikis- and epithelial‒mesenchymal transition-related signature for evaluating prognosis and predicting immunotherapy efficacy in gastric cancer
Source: J Cancer. 2025 Jan 6;16(4):1078–100. doi: 10.7150/jca.106029 (PMC11786038; doi:10.7150/jca.106029)
Supplement: Supplementary file 2 — Supplementary figures. [file jcav16p1078s2.pdf]

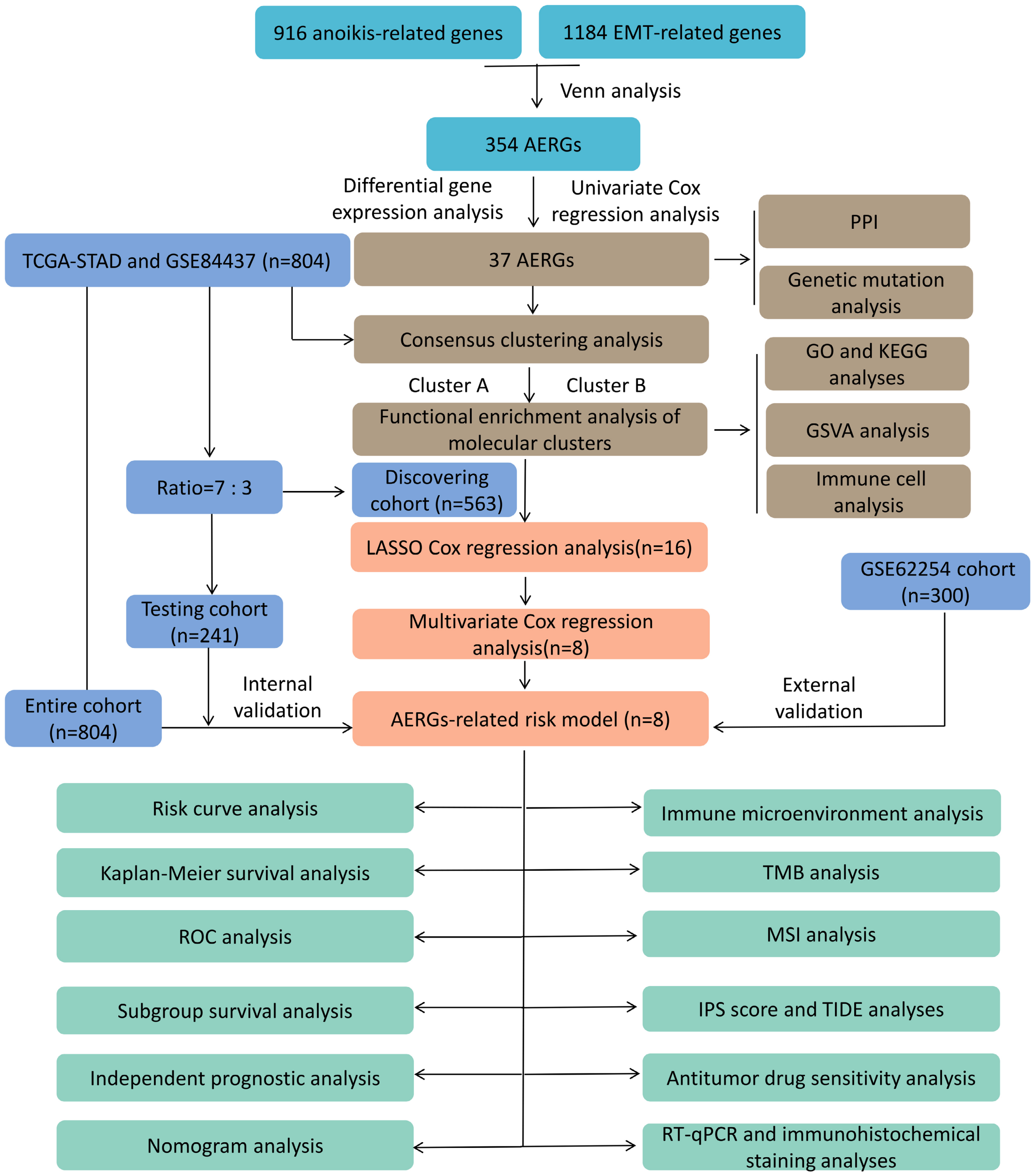

Figure S1 Flowchart of this study.

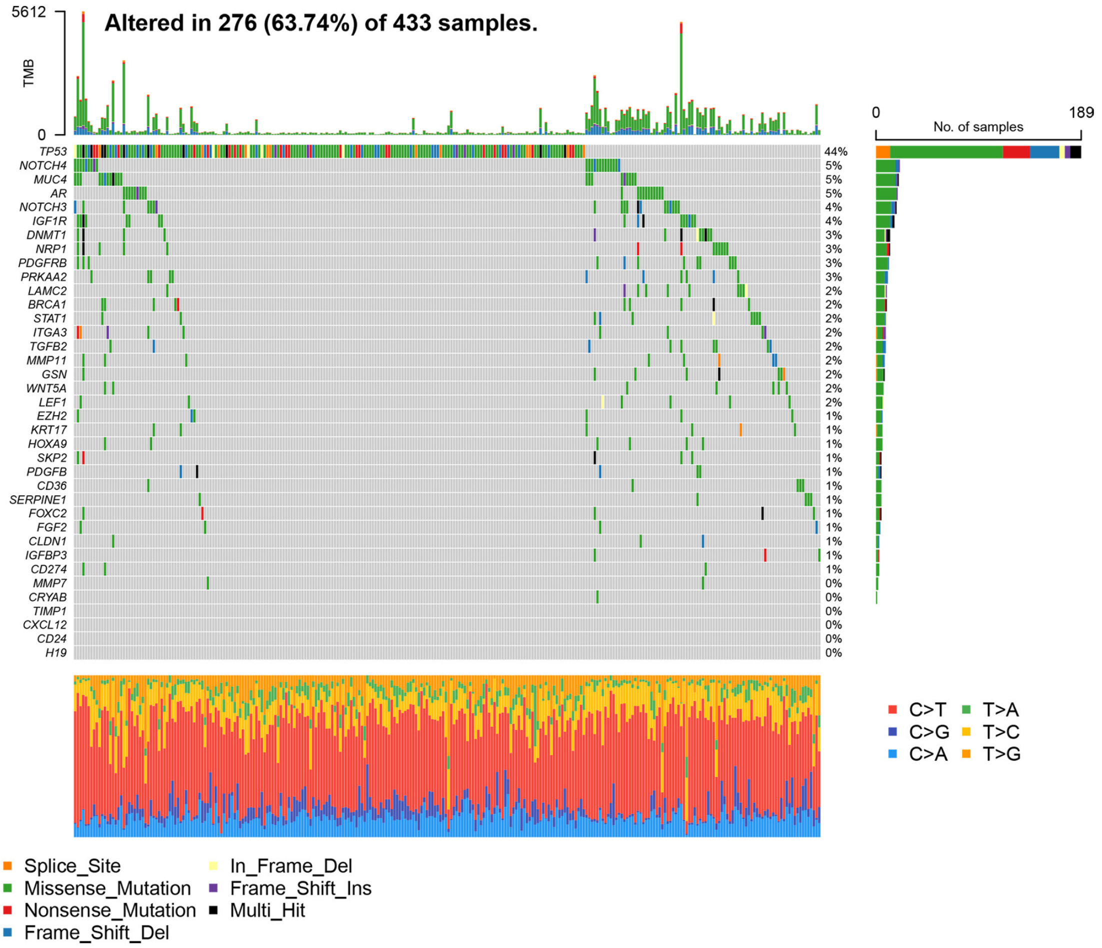

Figure S2 Mutational frequency analysis of AERGs in gastric cancer.

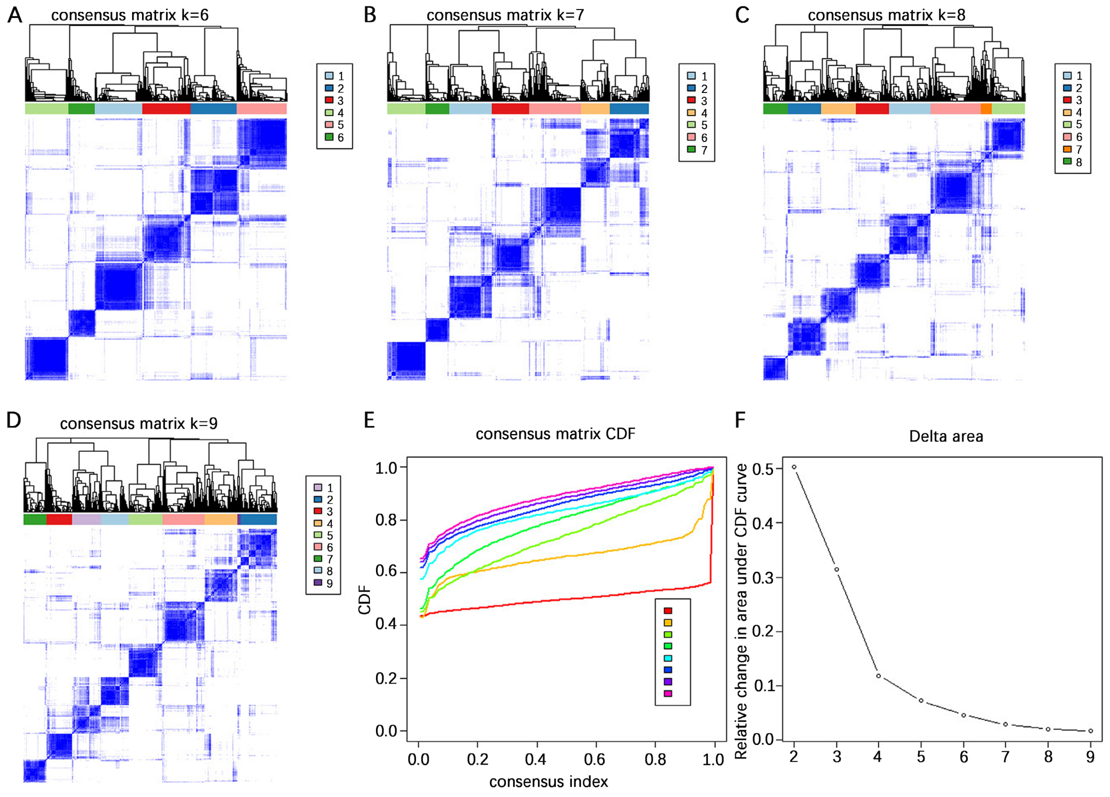

Figure S3 Consensus unsupervised clustering analysis of 37 AERGs in gastric cancer.

(A-D) Consensus matrix heatmaps of 37 AERGs (k = 6-9). **(E)** The CDF from k = 2 to 9. **(F)** Relative variation of the area under the CDF region from k = 2 to 9. CDF, cumulative distribution function.
